# Differentiated response mechanisms of soil microbial communities to nitrogen deposition driven by tree species variations in subtropical planted forests

**DOI:** 10.3389/fmicb.2025.1534028

**Published:** 2025-03-12

**Authors:** Zheng Hou, Wen Chen, Xiaohua Zhang, Donghui Zhang, Jinmei Xing, Yong Ba, Jie Yu, Keqin Wang, Ya Zhang, Yali Song

**Affiliations:** ^1^Kunming General Survey of Natural Resources Center, China Geological Survey, Kunming, China; ^2^College of Ecology and Environment, Southwest Forestry University, Kunming, China; ^3^Technology Innovation Center for Natural Ecosystem Carbon Sink, Ministry of Natural Resources, Kunming, China; ^4^Innovation Base for Eco-Geological Evolution, Protection and Restoration of Southwest Mountainous Areas, Geological Society of China, Kunming, China; ^5^School of Soil and Water Conservation, Beijing Forestry University, Beijing, China; ^6^College of Soil and Water Conservation, Southwest Forestry University, Kunming, China

**Keywords:** soil microbial diversity, richness, community structure, co-occurrence network, nitrogen deposition, planted forest

## Abstract

**Introduction:**

The increasing rate of atmospheric nitrogen deposition has severely affected the structure and function of these ecosystems. Although nitrogen deposition is increasing globally, the responses of soil microbial communities in subtropical planted forests remain inadequately studied.

**Methods:**

In this study, a four-year experimental simulation was conducted to assess the impacts of varying nitrogen deposition levels (CK: 0 g·N·m^−2^·a^−1^; N10: 10 g·N·m^−2^·a^−1^; N20: 20 g·N·m^−2^·a^−1^; N25: 25 g·N·m^−2^·a^−1^) on two subtropical tree species, *Pinus yunnanensis* Franch. and *Pinus armandii* Franch. High-throughput sequencing was performed using the Illumina MiSeq platform. Statistical analyses, including analysis of variance (ANOVA), linear mixed-effects models, principal coordinate analysis (PCoA), analysis of similarity (ANOSIM), redundancy analysis (RDA), random forest analysis, and structural equation modeling (SEM), were used to examine the short-term responses of soil nutrients, bacterial communities, and fungal community structures to nitrogen deposition.

**Results and discussion:**

The results showed that species differences led to variations in soil properties between the two forests, particularly a significant increase in soil pH in *P. yunnanensis* Franch. forests and a significant decrease in soil pH in *P. armandii* Franch. forests. Nitrogen addition did not significantly affect microbial diversity in either *P. yunnanensis* Franch. or *P. armandii* Franch. soils; however, forest type differences had a significant impact on bacterial diversity. The nitrogen addition significantly affected the relative abundance of specific microbial communities in both forest types, particularly altering the fungal community structure in the *P. yunnanensis* Franch forests, while no significant changes were observed in the bacterial community structure in either forest type. Furthermore, nitrogen addition increased the network complexity of bacterial communities in *P. yunnanensis* Franch. forests while decreasing network complexity in *P. armandii* Franch. forests. Structural equation modeling indicated that nitrogen addition regulates soil bacterial and fungal diversity in both forest types by modifying nitrogen availability.

**Purpose and significance:**

These findings provide insights into the potential long-term impacts of nitrogen deposition on subtropical planted forest ecosystems and offer a theoretical basis for sustainable forest management and regulatory practices.

## 1 Introduction

Human activities, such as fossil fuel combustion and agricultural practices, have significantly increased global nitrogen deposition, attracting considerable attention. From 2008 to 2020, the average total nitrogen (TN) deposition in China was 22.0 ± 0.8 kg·N·ha^−1^·yr^−1^ (Chen et al., 2023), ranking it as the third-largest region for nitrogen deposition worldwide. Recent studies indicate that although nitrogen deposition in China has stabilized, it remains elevated, with nitrogen deposition in some areas reaching 30–50 kg·N·ha^−1^·yr^−1^ (Zheng et al., 2022). Moderate nitrogen deposition can improve litter quality, enhance ecosystem productivity, and increase terrestrial carbon sink capacity (Lu et al., 2021). However, excessive deposition leads to nitrogen saturation in soils, reducing plant diversity, lowering the relative abundance and metabolic capacity of soil microbial communities, and causing other adverse effects (Janssens and Luyssaert, 2009; Kearns et al., 2016; Zhang et al., 2018). Soil microorganisms, as key components of ecosystems, play a crucial role in promoting key processes such as organic matter decomposition, carbon storage, and nutrient mineralization (Sokol et al., 2022; Kang et al., 2024). These processes are essential for maintaining soil health and functionality, directly shaping soil ecological functions. The global increase in nitrogen deposition is an important regulator of soil microbial communities, directly affecting microbial growth and turnover (Chen Y. et al., 2024).

Forest ecosystems are a crucial component of terrestrial ecosystems. The extensive surface area of forest canopies facilitates direct nitrogen deposition, while soil microbial communities beneath them, which occupy a substantial portion of the land surface, exhibit significant microbial diversity (Lladó et al., 2017) and are particularly sensitive to increased nitrogen deposition, especially elevated soil reactive nitrogen levels (Wang X. et al., 2023). Studies have shown that nitrogen deposition significantly alters soil parameters, leading to negative impacts on soil functions. For example, increased nitrogen deposition results in soil acidification, loss of nutrient-rich basic cations, and a reduction in nutrient utilization efficiency and buffering capacity. Additionally, nitrogen deposition may affect soil microbial dynamics by decreasing microbial biomass and altering microbial community structure through increased nitrogen availability. However, some studies have found that artificial nitrogen addition may lead to an increase in the abundance of key microbial groups, thereby promoting soil bacterial interactions and network complexity. This phenomenon may be linked to the complexity and heterogeneity of forest soils. Therefore, the impact of nitrogen deposition on microbial communities varies depending on the environmental conditions of forest soils, which limits the precision and applicability of research findings. Responses of microbial communities to nitrogen deposition vary with changes in overstory vegetation. Tree species associated with ectomycorrhizal fungi show improved survival and growth, while those linked to arbuscular mycorrhizal fungi exhibit reduced survival rates (Quinn Thomas et al., 2010). Variations in plant types alter soil physical and chemical properties, which act as filters for microbial community composition, influencing the diversity and richness of soil microbial communities (Lu et al., 2022). In ecosystems, microorganisms interact through direct or indirect ecological processes such as cooperation, competition, and antagonism, forming microbial networks (Wagg et al., 2019). Microbial co-occurrence networks provide insights into these complex interactions (Ramirez et al., 2018), where interspecies relationships often play a more critical role than species abundance and diversity (Deng et al., 2012). Nitrogen deposition significantly impacts soil functions by altering soil properties, with profound effects on microbial community structure and diversity. The type of vegetation and its interactions with microorganisms further exacerbate these effects. Studying the influence of nitrogen deposition on soil properties and microbial communities helps us better understand its long-term impact on forest ecosystems and provides a scientific basis for strategies aimed at improving soil health and maintaining ecological balance in forests.

The launch of the “United Nations Decade on Ecosystem Restoration” (Geekiyanage, 2022) has accelerated global afforestation efforts on degraded and deforested lands, generating significant environmental and societal benefits (Chazdon and Brancalion, 2019). In China, forest cover has increased from 13% in 1978 to 23% in 2019, with projections suggesting a rise to 26% by 2035 (Yao et al., 2024). This expansion has substantially enhanced the carbon sink capacity of terrestrial ecosystems. As a key nature-based solution, afforestation has been widely adopted to increase surface carbon storage, prevent soil erosion, conserve water resources, and produce timber, delivering critical ecosystem services and biodiversity gains (Hua et al., 2022). While considerable research has focused on the effects of nitrogen deposition on bacterial and fungal communities in forest ecosystems (Cao et al., 2023), studies addressing the responses of soil microbial communities in subtropical planted forests at low latitudes and high altitudes remain limited. Therefore, investigating the mechanisms underlying microbial responses to nitrogen input in planted forests under increasing global nitrogen deposition is essential for understanding the adaptive capacity and long-term ecological dynamics of these ecosystems in the context of global change.

This study employed a simulated nitrogen addition experiment at the Yuxi Forest Ecosystem National Observation and Research Station to explore the responses of soil microbial communities in *Pinus yunnanensis* Franch. and *Pinus armandii* Franch. plantations in the subtropical central Yunnan region to environmental changes and stress induced by long-term nitrogen addition. This research seeks to address the gap in studies on microbial responses to nitrogen deposition in the central Yunnan region. The hypotheses tested were: (1) Nitrogen addition decreases soil microbial diversity, with no significant effect of tree species differences on microbial diversity; (2) bacterial community structure responds significantly to nitrogen addition, while fungal communities show no significant response; and (3) changes in soil properties will drive shifts in both bacterial and fungal communities. Our findings provide microbiological insights for the scientific management of subtropical planted forests under nitrogen deposition and deepen our understanding of soil microbial functions and ecosystem processes in the context of global change.

## 2 Materials and methods

### 2.1 Study area overview

**Figure 1:**
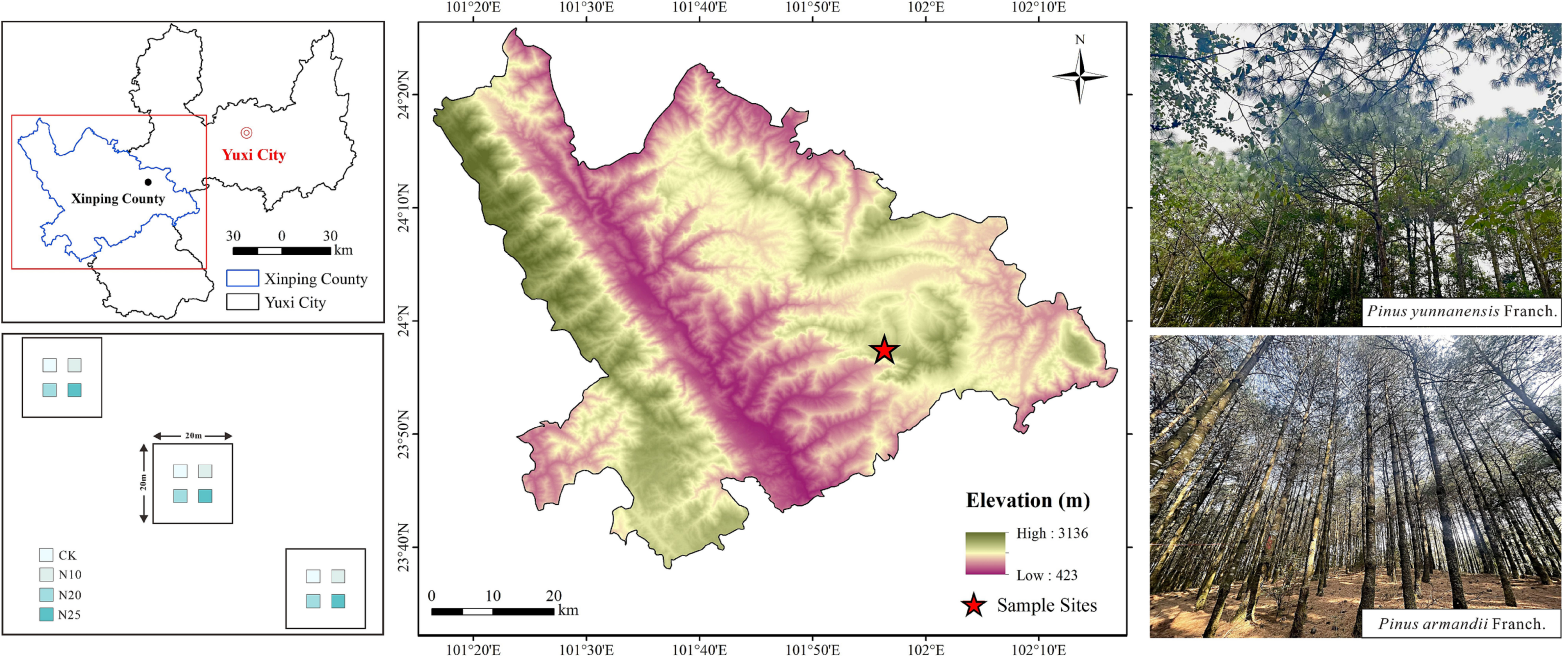
Study area location map.

This study was conducted at the Yuxi Forest Ecosystem National Observation and Research Station (23°46′18″ ~ 23°54′34″N, 101°16′06″ ~ 101°16′12″E) (Figure 1), where simulated nitrogen deposition began in January 2019. The station is situated in a climatic transition zone between northern and southern subtropical Yunnan, characterized by a subtropical low-latitude plateau monsoon climate with distinct mountain features. Elevations range from 1260.0 to 2614.4 m, with an average annual precipitation of 1,050 mm and an average annual temperature of 15°C. The region experiences temperature extremes of 33°C and −2.2°C, with distinct wet (May to October) and dry (November to April) seasons, where rain and heat coincide during the wet season. The primary soil type in the area is Argi-Udic Ferrosols, with localized occurrences of Hapli-Udic Argosols, as classified by the World Reference Base for Soil Resources. Soils are predominantly medium to thick, though thin layers are present in some areas. The forest coverage exceeds 86%, dominated by primary and secondary evergreen broadleaf forests typical of the montane semi-humid zone. Vegetation displays vertical zonation with altitude and includes species such as *P. yunnanensis* Franch., *P. armandii* Franch., *Keteleeria evelyniana* Mast., and plants from the *Magnoliaceae Juss.* family etc.

### 2.2 Plot design and sample collection

This study investigated two types of planted forests: *P. yunnanensis* Franch. forests and *P. armandii* Franch. forests. The *P. yunnanensis* Franch. forest is undergoing natural succession to increase species richness, while the species composition in the *P. armandii* Franch. forest is relatively simple. Through field surveys (Table 1), three representative 20 m × 20 m plots were selected in typical areas, with a minimum spacing of 10 m between plots. Each plot was subdivided into four 3 m × 3 m subplots using a split-plot design, separated by a 1 m buffer zone and walkway to prevent cross-treatment effects. Nitrogen addition levels were determined based on the atmospheric nitrogen deposition flux in Southwest China (Leng et al., 2018), the regional annual increase in nitrogen deposition (0.05 g·N·m^−2^·a^−1^) (Xing et al., 2024), seasonal deposition rates (15 g·N·m^−2^·a^−1^ in the dry season, 10 g·N·m^−2^·a^−1^ in the wet season) (Song et al., 2024), and future nitrogen deposition trends in the region. Four nitrogen addition treatments were established relative to the baseline annual nitrogen deposition of 3.84 g·N·m^−2^·a^−1^: 0 g·N·m^−2^·a^−1^ (CK), 10 g·N·m^−2^·a^−1^ (N10), 20 g·N·m^−2^·a^−1^ (N20), and 25 g·N·m^−2^·a^−1^ (N25). Experimental-grade urea [CO(NH₂)₂, ≥99.0%] was used as the nitrogen source. The total annual nitrogen addition for each treatment was divided into 12 equal portions, dissolved in 1 L of deionized water, and applied to the subplots monthly using a backpack sprayer. Control subplots received an equivalent volume of deionized water. Applications were conducted mid-month to ensure consistent treatment delivery.

**Table 1:** Site characteristics of two forest types.

Forest types	Stand	Altitude/m	Age/a	H/m	DBH/cm	Canopy density	Slope/(^o^)
*Pinus yunnanensis* Franch. Forest	1	2,193	25	8.7	10.5	0.73	13
	2	2,185	27	11.3	12.7	0.82	15
	3	2,236	25	9.4	11.3	0.75	12
*Pinus armandii* Franch. Forest	1	2,339	24	12.1	24.1	0.75	12
	2	2,337	23	11.0	17.0	0.79	13
	3	2,336	24	9.5	20.5	0.80	15

H, tree height; DBH, average diameter at breast height.

Soil samples were collected from the treatment subplots in *P. yunnanensis* Franch. and *P. armandii* Franch. forests on July 20, 2023 (during the wet season). During soil collection, surface litter was removed from the subplots. A five-point sampling method was employed, with a soil auger collecting samples from the 0–20 cm depth. Soil samples from three replicate subplots within the same treatment were thoroughly mixed and sieved through a 2 mm nylon mesh to remove roots and stones. The homogenized samples were then divided into two portions: one portion was stored at −80°C for high-throughput sequencing analysis, while the other portion was air-dried for soil property assessment.

### 2.3 Soil property measurements

Soil chemical properties were analyzed following the methods described by Bao (2000). TN was determined using the semi-micro Kjeldahl method. Available phosphorus (AP) was measured with inductively coupled plasma optical emission spectrometry (ICP-OES). Available potassium (K^+^) was determined using the flame photometric method with 1 mol·L^−1^ neutral NH₄OAc as the extractant. Soil organic matter (SOM) was calculated by first measuring organic carbon content using the K₂Cr₂O₇ heat-extraction method, then converting the value by multiplying the organic carbon content by a constant factor of 1.724. Soil ammonium nitrogen (NH₄^+^-N) was measured using the indophenol blue colorimetric method. Soil nitrate nitrogen (NO_3_^−^-N) was analyzed through ultraviolet spectrophotometry. Soil pH was assessed utilizing a glass electrode method with a soil-to-water ratio of 1:2.5 (*w*/*v*).

### 2.4 DNA extraction and microbial high-throughput sequencing

Microbial DNA from soil samples was extracted utilizing the E.Z.N.A™ Mag-Bind Soil DNA Kit (OMEGA Bio-tek, United States). The quantity of genomic DNA was measured with the Qubit^®^ 4.0 DNA quantification kit (Thermo Fisher Scientific, Shanghai, China) to ensure sufficient DNA for PCR amplification. Soil bacterial communities were analyzed by amplifying the V3–V4 region of the 16S rRNA gene using the primers 341F (5′-CCTACGGGNGGCWGCAG-3′) and 805R (5′-GACTACHVGGGTATCTAATCC-3′) (Logue et al., 2016). For fungal communities, the ITS region was amplified using the primers ITS1F (5′-CTTGGTCATTTAGAGGAAGTAA-3′) (Gardes and Bruns, 1993) and ITS2R (5′-GCTGCGTTCTTCATCGATGC-3′) (White et al., 1990). The purified and pooled PCR products were sequenced by Sangon Biotech (Shanghai) Co., Ltd. on the Illumina MiSeq platform for high-throughput sequencing.

### 2.5 Microbial data analysis and co-occurrence network construction

Raw sequence data underwent quality control and filtering before OTU clustering was performed using Usearch 11.0.667 software (Edgar, 2013) on non-redundant sequences (excluding singleton sequences), with a 97% similarity threshold for OTU definition. Chimera sequences were removed during clustering (Edgar et al., 2011). After OTU clustering, sequences were preprocessed by trimming adapter sequences and removing low-quality reads with FastQC (version 0.12.0) and Trimmomatic (version 0.39). Bacterial communities were classified using the SILVA database (version 132), and fungal communities were classified utilizing the UNITE database (version 8.0). Microbial community *α*-diversity and richness were calculated with Mothur 1.43.0 software, with the Shannon index used to quantify community diversity (Shannon, 1948) and the Chao index to estimate community richness (Chao, 1984). For OTU-based co-occurrence networks, OTUs with relative abundances above 0.1% in bacterial and fungal communities were included, while OTUs with zero abundance in two-thirds of the samples were excluded. Spearman correlation was used to calculate the pairwise similarity matrix, and Random Matrix Theory (RMT) determined the optimal similarity threshold for network construction. Molecular Ecological Networks (MENs) were analyzed using the Integrated Network Analysis Pipeline (iNAP) platform (updated on June 6, 2023, https://inap.denglab.org.cn/, accessed on July 18, 2024) (Feng et al., 2022). The node and edge data were then exported, and co-occurrence networks for bacterial and fungal communities were visualized with Gephi 0.10.1 software.

### 2.6 Data analysis methods

Statistical analysis was performed using SPSS 26.0 (SPSS Inc., Chicago, Illinois, United States). Analysis of variance (ANOVA) and Duncan’s test were employed to assess the effects of nitrogen treatments on soil chemical properties and microbial *α*-diversity. A linear mixed-effects model was applied to evaluate nitrogen addition effects, using tree species as a random factor and nitrogen addition as a fixed factor. Tukey’s Honest Significant Difference (HSD) test was applied at a significance level of 0.05 if significant effects were detected. In R software (version 4.3.1; http://www.r-project.org/, accessed July 20, 2024), the “vegan” (Oksanen et al., 2013) and “ggplot2” (Wickham et al., 2016) packages were used for principal coordinates analysis (PCoA) and analysis of similarities (ANOSIM) based on microbial genus-level classification. Bray–Curtis dissimilarity was calculated to assess differences in microbial community structure across nitrogen addition treatments. Redundancy analysis (RDA) was also performed to identify key soil properties influencing bacterial and fungal community composition in forest soils. The “random Forest” package (R ColorBrewer and Liaw, 2018) was employed to evaluate the importance of soil chemical properties in shaping microbial community composition, with the first axis of PCoA representing community composition. Structural equation modeling (SEM) was conducted utilizing the “lavaan” package (Svetina et al., 2020) to assess the impact of nitrogen addition and soil properties (NO_3_^−^-N, NH_4_^+^-N, K^+^, pH) on microbial communities. The chi-square test (*χ*^2^) was used to assess the overall model fit by comparing the model-predicted covariance matrix to observed data. Model significance was assessed using the *p*-value, and the root mean square error of approximation (RMSEA) was used to evaluate model-data fit and overall goodness of fit.

## 3 Results and analysis

### 3.1 Response of soil basic properties to nitrogen addition

**Figure 2:**
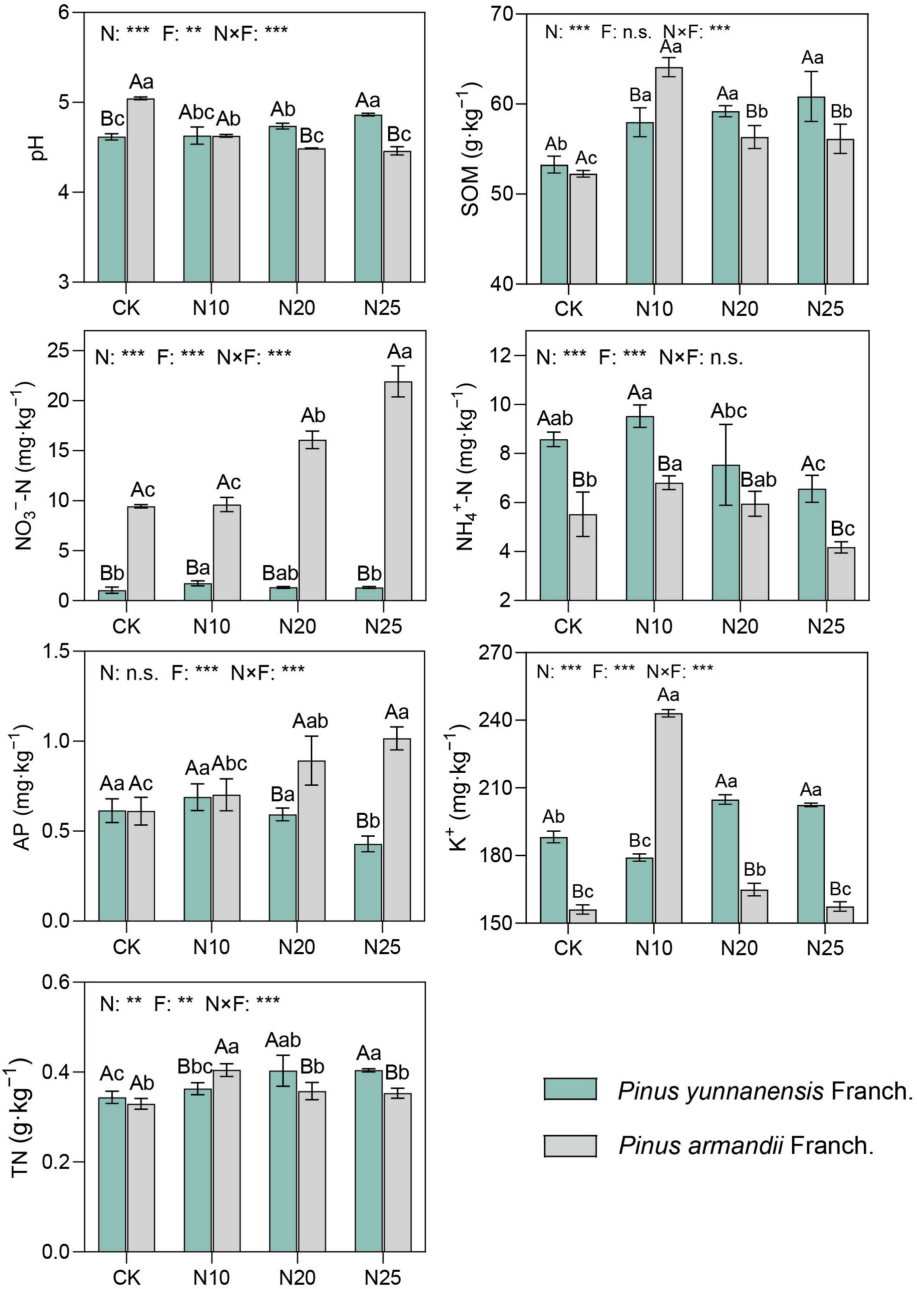
Characteristics of soil properties under different levels of nitrogen deposition. (1) Lower case letters indicate significant differences between nitrogen deposition treatments and uppercase letters indicate significant differences between forest types. (2) n.s. indicates *p* > 0.05, * indicates *p* < 0.05, ** indicates *p* < 0.01, *** indicates *p* < 0.001. (3) CK, 0 g·N·m^−2^·a^−1^; N10, 10 g·N·m^−2^·a^−1^; N20, 20 g·N·m^−2^·a^−1^; and N25, 25 g·N·m^−2^·a^−1^. SOM, soil organic matter; TN, total nitrogen; AP, available phosphorus; K^+^, potassium ion.; N, nitrogen deposition; F, forest types; N × F, nitrogen deposition and forest type interactions.

Compared to the control (CK) of the two stands, the soil NH_4_^+^-N and K^+^ contents in the *P. yunnanensis* Franch. forest control group were significantly higher than those in the *P. armandii* Franch. Forest control group, with increases of 55.4 and 20.6%, respectively, while the pH and NO_3_^−^-N contents in the *P. yunnanensis* Franch. forest were lower than those in the *P. armandii* Franch. forest, by 8.5 and 88.9%, respectively (Figure 2). In *P. yunnanensis* Franch. forests, with increasing nitrogen addition, soil pH and SOM gradually increased, with increases of 0.2–5.2% and 8.8–14.2%, respectively. The contents of NH_4_^+^-N, NO_3_^−^-N, and AP initially increased and then decreased. In *P. armandii* Franch. forests, with increasing nitrogen addition, soil pH gradually decreased, with a reduction of 8.1–13.0%. SOM, NH_4_^+^-N, K^+^, and TN increased initially and then decreased, while NO_3_^−^-N and AP contents significantly increased, with increases of 1.9–132.2% and 15.1–66.3%, respectively. Overall, nitrogen addition significantly influenced soil chemical properties in both forest types, though the patterns of change differed. These results suggest that the impact of nitrogen addition on soil basic properties varies with forest type, reflecting the differential responses of the two tree species to nitrogen addition. Overall, the results demonstrate that nitrogen addition significantly affects the soil chemical properties of both forest types. However, the impact varies between the two types, indicating that different forest ecosystems respond differently to nitrogen inputs. These findings underscore the complex interactions between nitrogen deposition and soil properties, highlighting the importance of considering forest type when evaluating the ecological consequences of nitrogen addition.

### 3.2 Response of soil microbial diversity to nitrogen addition

**Figure 3:**
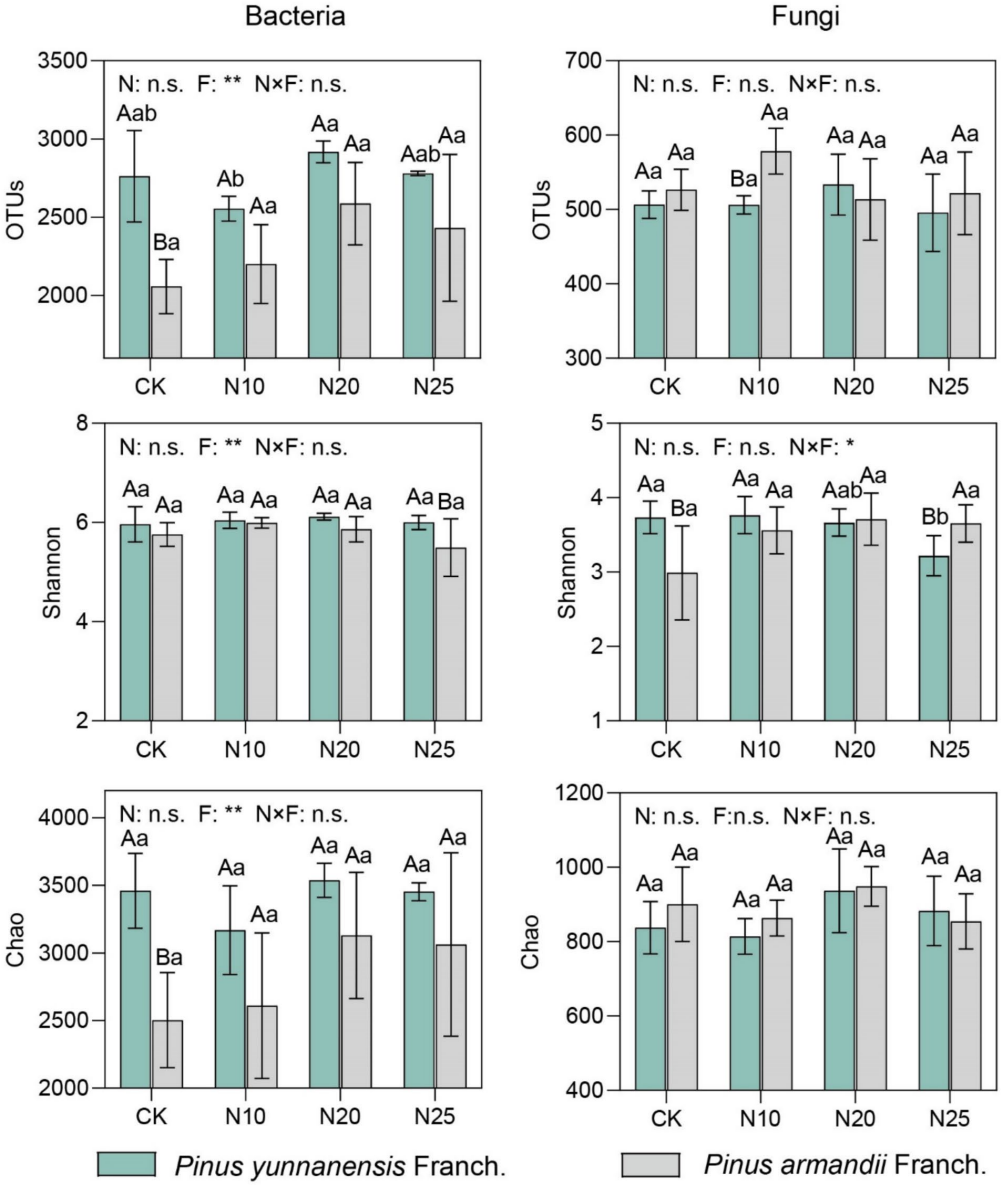
Analysis of alpha diversity in soil bacterial and fungal communities. (1) Lower case letters indicate significant differences between nitrogen deposition treatments and uppercase letters indicate significant differences between forest types. (2) n.s. indicates *p* > 0.05, * indicates *p* < 0.05, ** indicates *p* < 0.01, *** indicates *p* < 0.001. (3) CK, 0 g·N·m^−2^·a^−1^; N10, 10 g·N·m^−2^·a^−1^; N20, 20 g·N·m^−2^·a^−1^; and N25, 25 g·N·m^−2^·a^−1^. N, nitrogen deposition; F, forest types; N × F, nitrogen deposition and forest type interactions.

Soil microbial α-diversity, including OTUs, Shannon index, and Chao index, was used to evaluate the impact of nitrogen addition on the diversity and richness of bacterial and fungal communities in *P. yunnanensis* Franch. and *P. armandii* Franch. forests (Figure 3). Compared to the CK, the soil of *P. yunnanensis* Franch. exhibited higher bacterial community OTUs, Shannon index, and Chao index than that of *P. armandii* Franch., with increases of 34.2, 3.5, and 38.2%, respectively. In contrast, fungal OTUs and the Chao index were lower by 3.8 and 7.0%, respectively, while the fungal Shannon index was 24.7% higher than that of *P. armandii* Franch. In *P. yunnanensis* Franch. forests, the soil bacterial community OTUs, Shannon index, and Chao index all reached their peaks under the N20 treatment, showing increases of 5.5, 2.7, and 2.2%, respectively, compared to the CK. This suggests that moderate nitrogen addition promotes bacterial diversity. However, at the highest nitrogen addition level (N25), bacterial OTUs, Shannon index, and Chao index decreased by 4.9, 2.0, and 2.4%, respectively, indicating that high nitrogen concentrations may suppress bacterial diversity. In *P. armandii* Franch. forests, there were no significant differences in bacterial community diversity and richness across different nitrogen treatments, but overall, OTUs and Chao index increased, with the N20 treatment showing the highest increases of 25.7 and 25.0%, respectively, while the Shannon index gradually decreased. On the other hand, for the fungal community, nitrogen addition generally decreased the OTUs and Shannon index in *P. armandii* Franch. forests, with the most notable decreases of 2.1 and 13.7% under the N25 treatment, whereas the Chao index increased by 5.4%, suggesting that high nitrogen concentrations may have a negative impact on fungal community diversity. In *P. armandii* Franch. forests, fungal communities exhibited a minimal response to nitrogen addition, with no significant changes in *α*-diversity indices across treatments. However, the Shannon index and Chao index peaked at the N20 treatment. Overall, nitrogen addition influenced soil bacterial and fungal diversity more significantly in *P. yunnanensis* Franch. forests, while the impact on *P. armandii* Franch. forests was smaller and not statistically significant. Statistical analysis indicated that forest type significantly affected bacterial diversity indices (*p* < 0.05), but no significant interaction was observed between nitrogen addition and forest type for most diversity indices.

**Figure 4:**
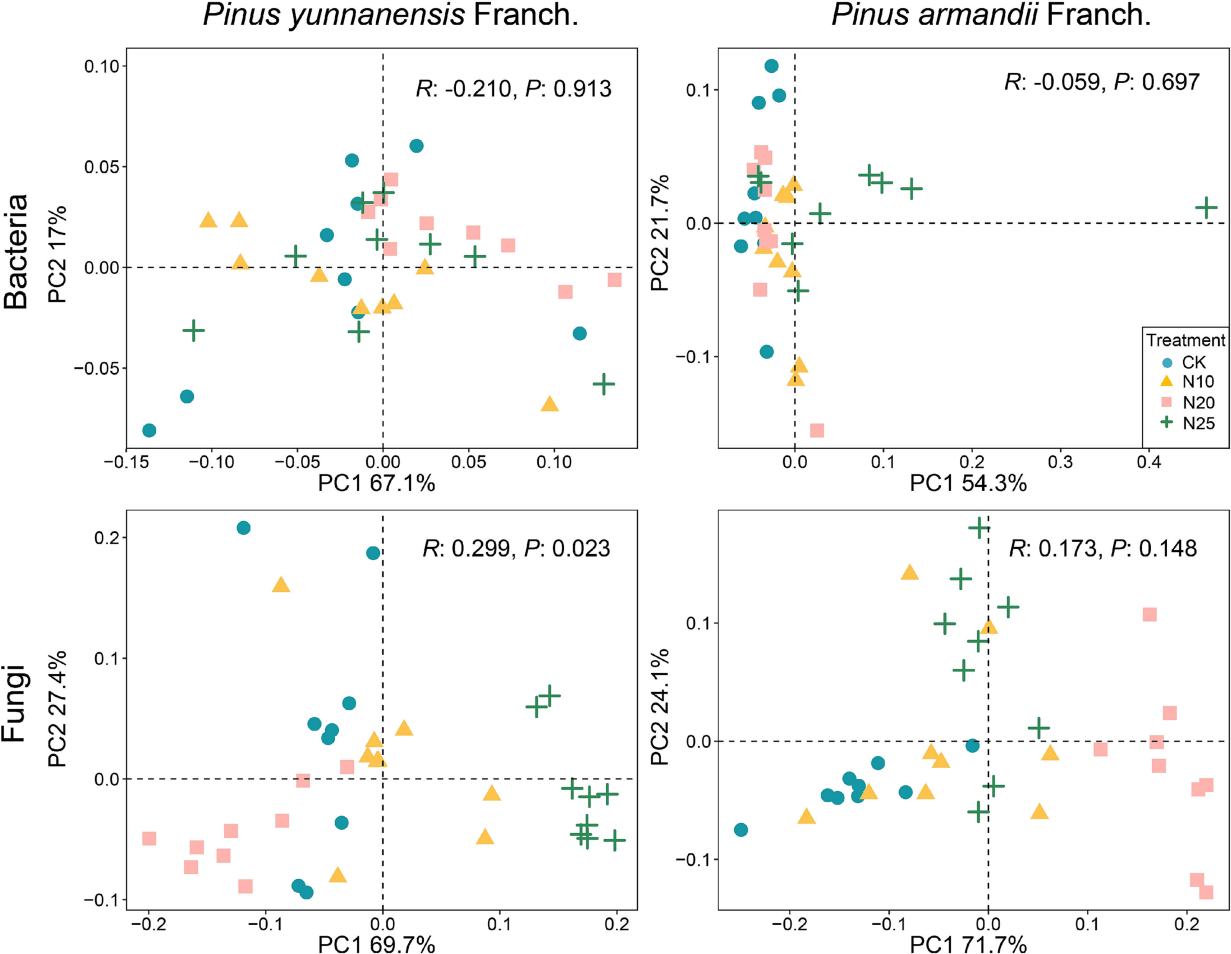
Principal coordinate analysis (PCoA) was performed to analyze the bacterial and fungal communities in across different forest stands subjected to various nitrogen addition levels. CK, 0 g·N·m^−2^·a^−1^; N10, 10 g·N·m^−2^·a^−1^; N20, 20 g·N·m^−2^·a^−1^; and N25, 25 g·N·m^−2^·a^−1^.

Principal coordinates analysis was conducted to evaluate the β-diversity of soil bacterial and fungal communities in *P. yunnanensis* Franch. and *P. armandii* Franch. forests under different nitrogen addition treatments (Figure 4). The results showed that nitrogen addition significantly altered the fungal community structure in *P. yunnanensis* Franch. forests (*R*: 0.299, *P*: 0.023), while the fungal community structure in *P. armandii* Franch. forests was also affected, though the changes were not statistically significant. Additionally, the bacterial community structure in both forest types showed no significant changes under nitrogen addition, indicating differing sensitivities among microbial communities. These results suggest that fungal communities are more sensitive to nitrogen addition, while bacterial communities may possess greater adaptability to environmental changes.

### 3.3 Effect of nitrogen addition on microbial community composition

**Figure 5:**
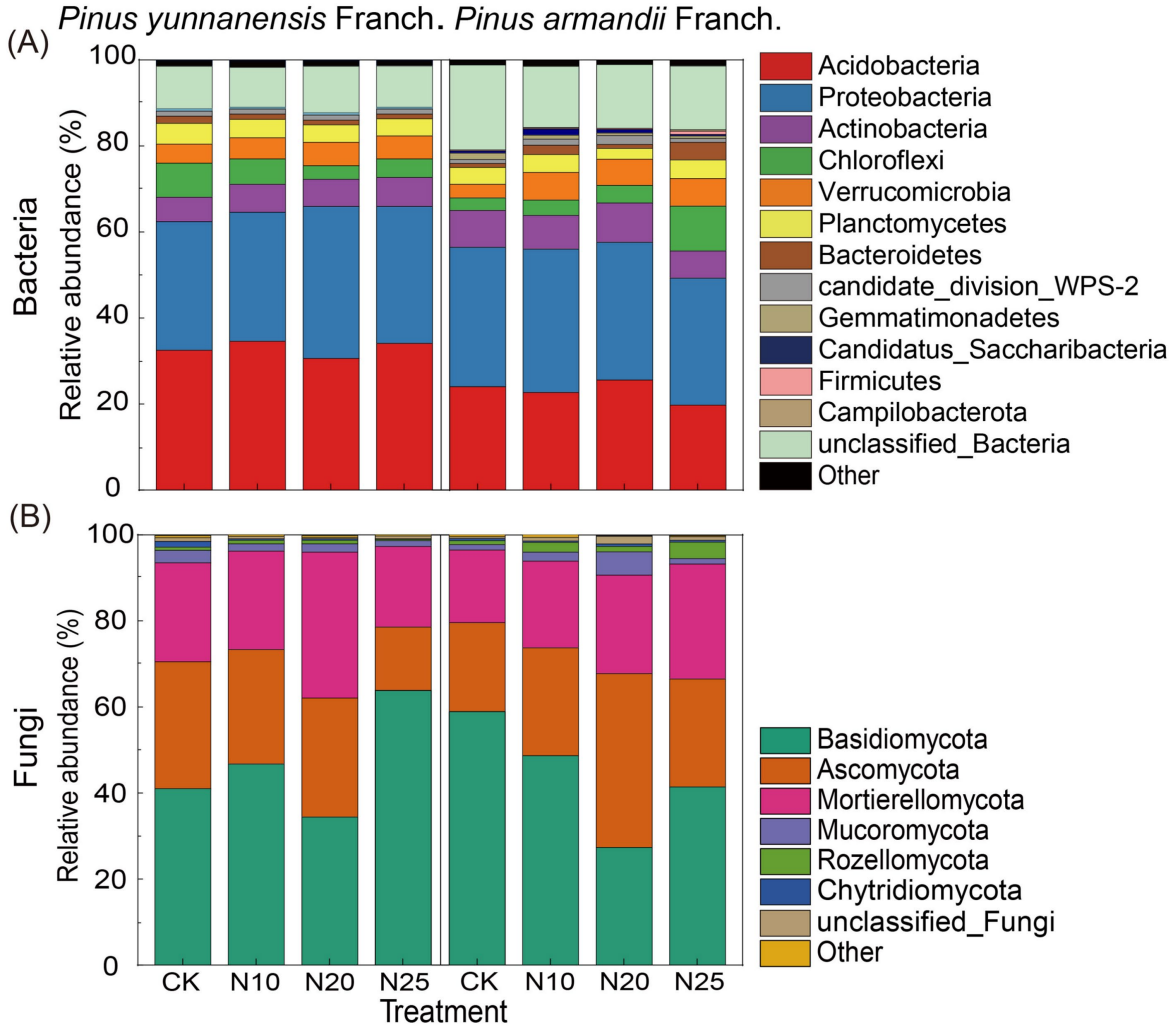
The relative abundance of bacterial and fungal phyla in the two forest stands. Relative abundance of bacterial phyla **(A)**; relative abundance of fungal phyla **(B)**. CK, 0 g·N·m^−2^·a^−1^; N10, 10 g·N·m^−2^·a^−1^; N20, 20 g·N·m^−2^·a^−1^; and N25, 25 g·N·m^−2^·a^−1^.

Figure 5 shows the impact of nitrogen addition on microbial community composition at the phylum level. In the soil of *P. yunnanensis* Franch. forests, 11 bacterial phyla and 8 fungal phyla were identified, while in the soil samples of *P. armandii* Franch. forests, 14 bacterial phyla and 8 fungal phyla were identified. Notably, the dominant bacterial phyla in both *P. yunnanensis* Franch. and *P. armandii* Franch. forest soils were *Acidobacteria* and *Proteobacteria*, accounting for 45.44 to 67.92% of the total bacterial community (Figure 5A). Similarly, *Basidiomycota* and *Ascomycota* were the dominant fungal phyla, representing approximately 61.58 to 80.29% of the total fungal community (Figure 5B). The relative abundance of microbial taxa showed no significant differences between the two forest types.

As shown in Supplementary Table S1, nitrogen addition did not significantly affect the overall relative abundance of bacterial phyla in *P. yunnanensis* Franch. soils. However, as nitrogen levels increased, the relative abundance of *Proteobacteria*, *Verrucomicrobia*, *candidate_division_WPS-2*, *Candidatus_Saccharibacteria*, and *unclassified Bacteria* first increased and then decreased, peaking at the N20 treatment. The relative abundance of *Planctomycetes* and *Bacteroidetes* gradually decreased. *Basidiomycota* abundance significantly increased 58.2% in the fungal community under high nitrogen levels (N25), while other fungal phyla displayed no significant changes. In contrast, *Ascomycota* abundance decreased progressively as nitrogen levels increased. Moreover, correlation analysis (Supplementary Table S2) indicated that SOM was positively associated with *Verrucomicrobia* but negatively associated with *Bacteroidetes* and *Chytridiomycota*. Soil pH showed a positive correlation with *Basidiomycota*. NO_3_^−^-N was negatively correlated with *Chloroflexi* and *Mucoromycota* but positively correlated with *other* microbial taxa.

In *P. armandii* Franch. forests, nitrogen addition significantly influenced the *Gemmatimonadetes* abundance in the soil bacterial community, which gradually decreased with increasing nitrogen levels, with a reduction ranging from 55.3 to 198.0% (Supplementary Table S3). Additionally, nitrogen addition decreased the *Proteobacteria* and *unclassified Bacteria* abundance while increasing *Chloroflexi* and *Firmicutes*, although no significant differences were observed between different nitrogen levels. In the soil fungal community, nitrogen addition significantly reduced the relative abundance of *Basidiomycota* by 17.9 to 105.9% compared to the CK, while the relative abundance of *Ascomycota* significantly increased by 12.0 to 91.9%. Furthermore, the relative abundance of *Mortierellomycota* and *Rozellomycota* increased, though these changes were not significant. Correlation analysis (Supplementary Table S4) showed that *Gemmatimonadetes* was positively correlated with pH and negatively correlated with NO_3_^−^-N and AP. Basidiomycota showed a significant positive link with pH, while *Rozellomycota* showed negative correlations with pH but positive correlations with NO_3_^−^-N and AP.

### 3.4 Soil microbial co-occurrence network

**Figure 6:**
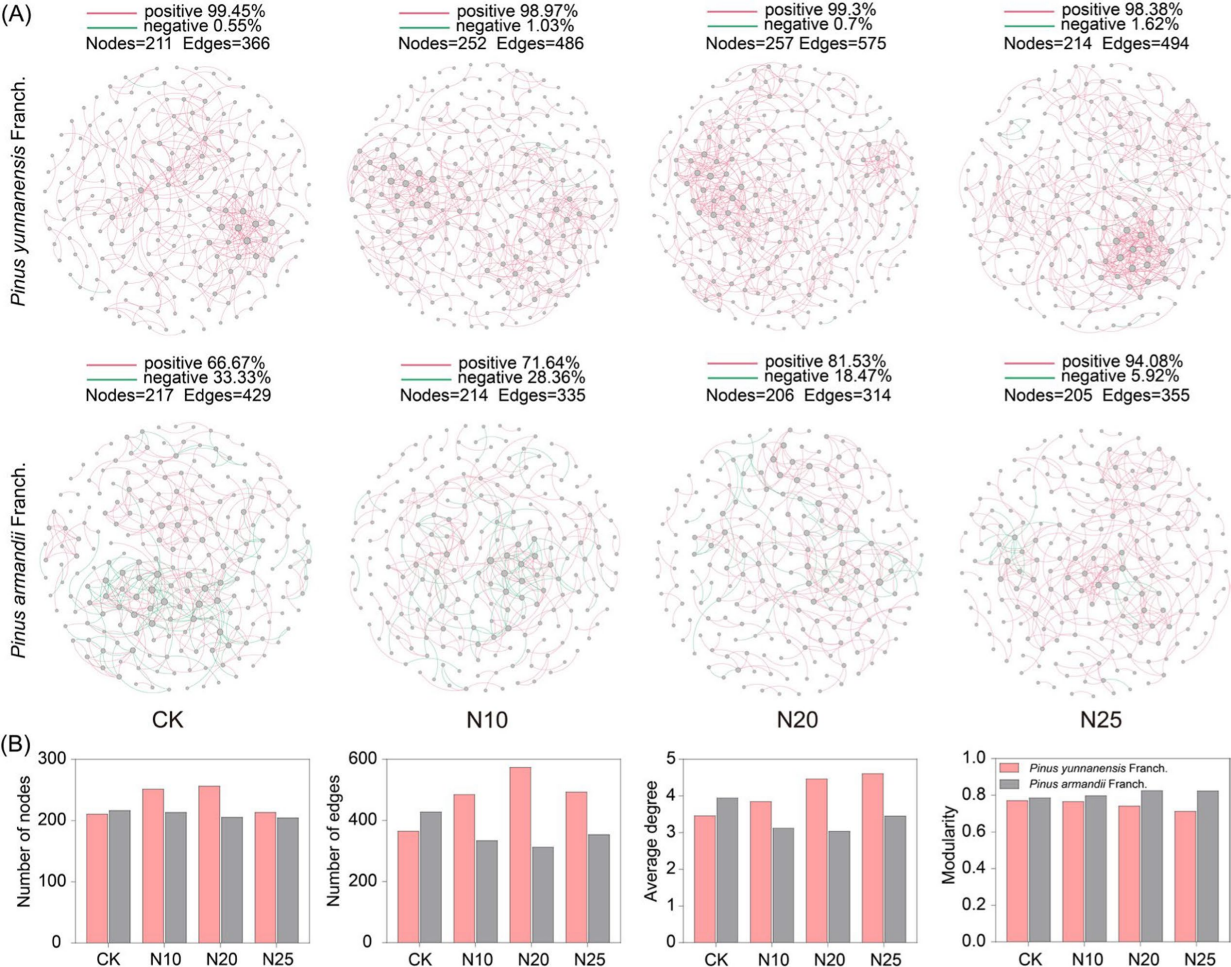
Soil bacterial co-occurrence network-based OTU profile **(A)**. Characterization of the properties of bacterial co-occurrence networks **(B)**. CK, 0 g·N·m^−2^·a^−1^; N10, 10 g·N·m^−2^·a^−1^; N20, 20 g·N·m^−2^·a^−1^; and N25, 25 g·N·m^−2^·a^−1^. Node size indicates the connection size of the module, red connecting lines indicate cooperative relationships between species, and green connecting lines indicate competitive relationships.

Microbial co-occurrence networks were constructed using Random Matrix Theory (RMT) to analyze bacterial and fungal community interaction changes under different nitrogen addition levels. As shown in Figure 6, compared to CK, the number of nodes in the co-occurrence networks of both *P. yunnanensis* Franch. and *P. armandii* Franch. forests did not exhibit significant differences compared to the control (CK). However, the number of connections (edges) in the bacterial co-occurrence network of *P. armandii* Franch. forest was 17.2% higher than that of *P. yunnanensis* Franch. forest, indicating a higher complexity in the bacterial species interactions in the *P. armandii* Franch. forest. In *P. yunnanensis* Franch. forest, bacterial co-occurrence network characteristics, including the number of nodes, connections, and average degree, increased with nitrogen addition. However, the modularity value decreased as nitrogen levels increased, while the ratio of positive correlation edges remained stable. In *P. armandii* Franch. forest, the bacterial co-occurrence network showed a declining trend in the number of nodes, connections, and average degree with increasing nitrogen addition, reaching the lowest values at the N20 treatment. The modularity value increased with nitrogen levels, and the ratio of positive correlation increased significantly. These results suggest that in the *P. yunnanensis* Franch. forest, the complexity of bacterial species interactions increased with nitrogen addition, peaking at the N20 treatment, while in the *P. armandii* Franch. forest, the complexity of bacterial species interactions decreased with increasing nitrogen levels.

**Figure 7:**
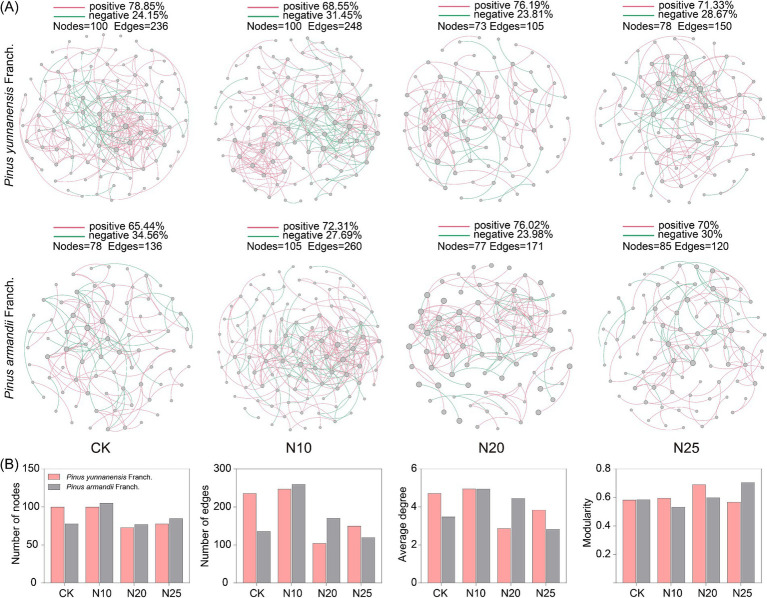
Soil fungal co-occurrence network-based OTU profile **(A)**. Characterization of the properties of fungal co-occurrence networks **(B)**. CK, 0 g·N·m^−2^·a^−1^; N10, 10 g·N·m^−2^·a^−1^; N20, 20 g·N·m^−2^·a^−1^; and N25, 25 g·N·m^−2^·a^−1^. Node size indicates the connection size of the module, red connecting lines indicate cooperative relationships between species, and green connecting lines indicate competitive relationships.

Similarly, compared to CK (Figure 7), the fungal co-occurrence network in *P. yunnanensis* Franch. forest had significantly more nodes and edges compared to *P. armandii* Franch. forest, suggesting greater fungal species interaction complexity in the former. Forest. After nitrogen addition, the network characteristics of the fungal co-occurrence network in the *P. yunnanensis* Franch. forest, including the number of nodes, connections, and average degree, initially increased and then decreased, peaking at the N10 treatment. In *P. armandii* Franch. forest, the fungal co-occurrence network characteristics, including the number of nodes, connections, and average degree, all decreased with increasing nitrogen addition, while the modularity value gradually increased. These findings suggest that high nitrogen addition (N25) suppressed fungal network complexity and stability, while low nitrogen addition (N10) enhanced fungal species interactions in *P. yunnanensis* Franch. forest.

### 3.5 Soil microbial co-occurrence network

**Figure 8:**
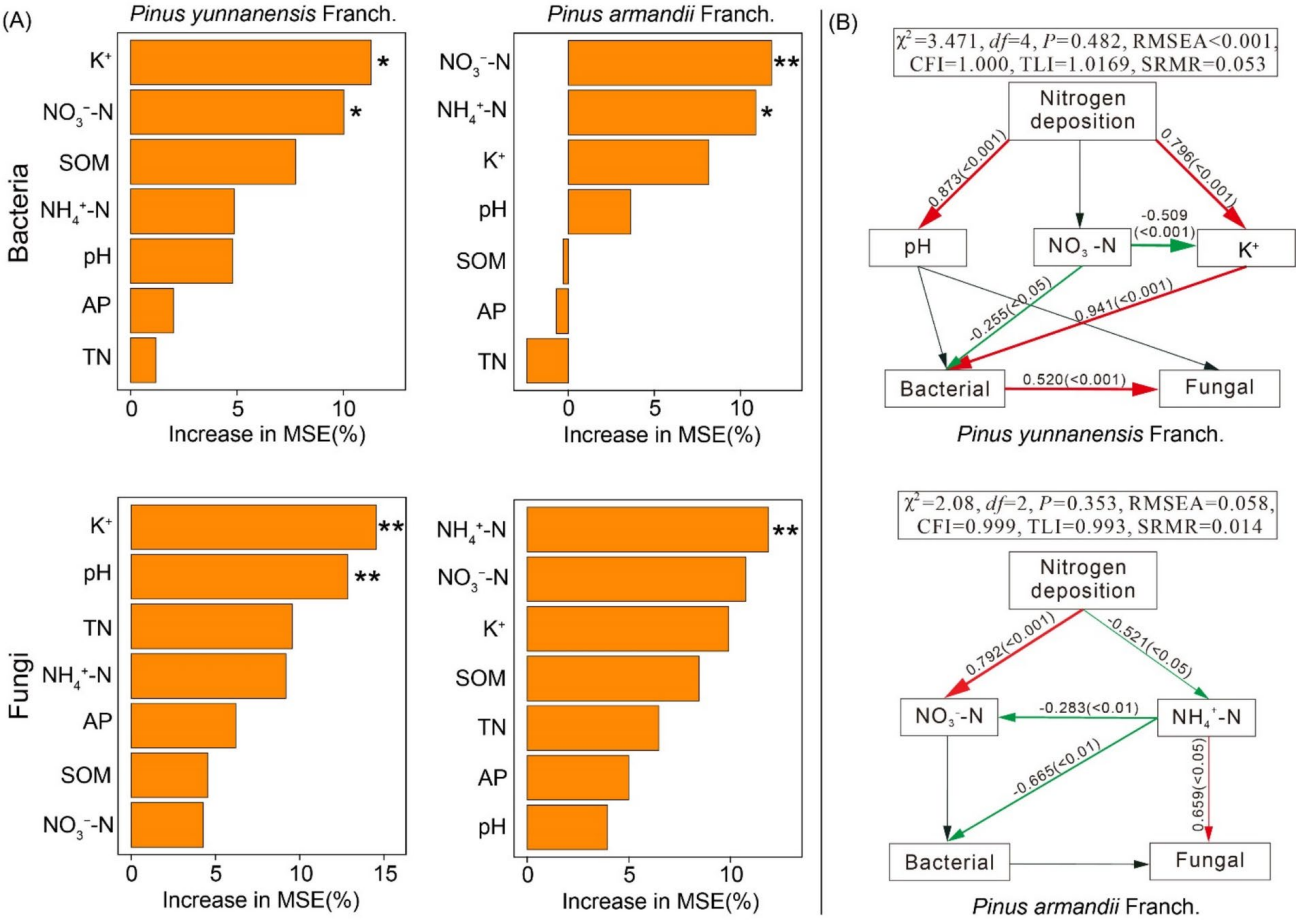
Soil properties are key drivers of variations in soil bacterial and fungal community structure and diversity. The importance of each variable was assessed using random forests **(A)**. Structural equation modeling (SEM) was employed to analyze soil-microbial community interactions in response to nitrogen addition **(B)**. * indicates *p* < 0.05, ** indicates *p* < 0.01; pH, pondus hydrogenii; SOM, soil organic matter; NH_4_^+^-N, soil ammonium N; NO_3_^−^-N, soil nitrate N; TN, total nitrogen; AP, available phosphorus; K^+^, available potassium; Red arrows indicate significant positive correlations (*p* < 0.05), green arrows indicate significant negative correlations (*p* < 0.05), and gray arrows indicate non-significant relationships (*p* > 0.05).

Soil properties play a crucial role in shaping differences in bacterial and fungal community structures across forest types. Redundancy Analysis (RDA) demonstrated that environmental factors explained 72.31 and 63.78% of the variation in soil bacterial community structure in *P. yunnanensis* Franch. and *P. armandii* Franch. forests, respectively, and 79.38 and 74.69% of the variation in fungal community structure (Supplementary Figure S1). Additionally, Random Forest analysis identified key soil variables influencing microbial community structure, while SEM quantified the direct and indirect effects of these factors on microbial community composition. The Random Forest analysis revealed that K^+^, NO_3_^−^-N, and pH were the primary variables determining bacterial and fungal community composition and richness in *P. yunnanensis* Franch. forest, while NO_3_^−^-N and NH_4_^+^-N were the primary variables influencing microbial community structure in the *P. armandii* Franch. forest (Figure 8A). The Random Forest model was also used to investigate the impact of nitrogen addition on microbial diversity (Figure 8B). The model for the *P. yunnanensis* Franch. forest showed good data fit (*χ*^2^ = 3.471, *df* = 4, *p* = 0.482, RMSEA <0.001), as did the model for *P. armandii* Franch. forests (*χ*^2^ = 2.08, *df* = 2, *p* = 0.353, RMSEA = 0.058). In the *P. yunnanensis* Franch. forest model, soil NO_3_^−^-N had a significant negative effect on bacterial *α*-diversity, while K^+^ had a significant positive effect on bacterial α-diversity. Additionally, the model showed a significant positive interaction between bacteria and fungi. In the *P. armandii* Franch. forest model, NH_4_^+^-N negatively impacted bacterial α-diversity but positively affected fungal α-diversity. These findings suggest that nitrogen addition impacts microbial communities differently across forest types. Specifically, in the *P. yunnanensis* Franch. forest, soil NO_3_^−^-N and K^+^ are key drivers of bacterial α-diversity, whereas in the *P. armandii* Franch. forest, NH_4_^+^-N is the primary factor influencing both bacterial and fungal α-diversity.

## 4 Discussion

### 4.1 Effects of nitrogen addition on soil microbial diversity and community structure

In our study, continuous nitrogen addition did not significantly reduce soil microbial diversity in both forest types, this is contrary to our hypothesis (1). However, differences in soil background properties caused by tree species led to a significant response of soil microbial diversity to nitrogen addition. Studies have confirmed that soil pH can lead to variations in soil resistance and stability (Tian and Niu, 2015), and tree species characteristics play an important role in influencing soil pH (Finzi et al., 1998). In this study, soil pH varied significantly between the two forest types (*P. yunnanensis* Franch. pH ranged from 4.62 to 4.86; *P. armandii* Franch. pH ranged from 5.04 to 4.46), reflecting distinct response mechanisms to nitrogen addition. Research has shown that the balance between basic cations (e.g., K^+^, Ca^2+^, Mg^2+^) and acidic cations (H^+^) governs soil pH changes (Berthrong et al., 2009; Lu et al., 2014). In *P. yunnanensis* Franch. forests, nitrogen addition increased soil pH, accompanied by fluctuating K^+^ levels, suggesting strong buffering capacity that neutralized H^+^ and mitigated acidification. In contrast, in the *P. armandii* Franch. forest, nitrogen addition led to a reduction in pH, and K^+^ content gradually decreased with increasing nitrogen addition, reflecting weaker buffering capacity and reduced resistance to acidification. This difference may be related to the initial soil conditions in the two forest types and their distinct response mechanisms to nitrogen addition. Additionally, the significant reduction in pH in the *P. armandii* Franch. forest may result from nitrogen addition enhancing nitrification. Typically, NH_4_^+^-N is the dominant form of inorganic nitrogen input, while NO_3_^−^-N is the primary form of nitrogen output (Rosenstock et al., 2019). During soil nitrogen cycling, the oxidation of NH_4_^+^ to NO_3_^−^ through nitrification is a major acid-producing process. The significant decrease in NH_4_^+^-N and increase in NO_3_^−^-N in the *P. armandii* Franch. forest suggests active nitrification, generating large amounts of H^+^ and increasing soil acidity. Urea, used as the nitrogen source in this study, has a relatively low soil acidification potential (Wang Z. et al., 2023). During the wet season, urea hydrolysis consumes H^+^, reducing net H^+^ concentrations in both *P. armandii* Franch. and *P. yunnanensis* Franch. forests. In comparison, *P. yunnanensis* Franch. preferentially absorbs ammonium (NH_4_^+^), leading to significant uptake of NH₄^+^ by the dominant plant species (Kronzucker et al., 1997; Tian et al., 2020), significantly reducing the NH₄^+^ available for soil nitrification. Although some NH_4_^+^ in the *P. yunnanensis* Franch. Forest undergoes nitrification, and the limited availability of nitrification substrates reduces H^+^ production, lowering the potential for soil acidification. The observed increase in pH in the *P. yunnanensis* Franch. forest may be attributed to this phenomenon.

Previous studies have shown that nitrogen addition generally decreases the α-diversity of soil bacteria and fungi in forest ecosystems (Allison et al., 2007; Nie et al., 2018; Wang X. et al., 2023), primarily due to nitrogen-induced soil acidification, which leads to a decrease in root biomass and root exudation, as well as a reduction in soil microbial biomass (Yang et al., 2022). However, after 4 years of nitrogen addition, no significant impact on the α-diversity of soil bacteria and fungi was observed in our study (Figure 3). Short-term nitrogen addition does affect microbial communities, but these effects are transient and disappear after longer periods of nitrogen addition (>4 years) (Liu et al., 2013). The duration of nitrogen addition appears to be a key factor. Meanwhile, studies have suggested that water availability can directly affect soil microorganisms (Chen W. et al., 2024). Increased rainfall during the wet season enhances the positive effects of nitrogen addition on microbial communities, as water availability creates favorable conditions for microbial growth. The interaction between water availability and nitrogen addition provides favorable conditions for microbial growth, shifting the effects from negative to positive (Yang et al., 2024). The mitigating effects of the wet season offset the negative impacts of short-term nitrogen addition, which explains the absence of significant changes in soil bacterial and fungal diversity in the study. Forest type significantly influenced soil bacterial diversity, likely due to the indirect effects of dominant tree species and structural differences on microbial communities through variations in the quantity and quality of plant-derived carbon inputs (e.g., root exudates, litter) (Terrer et al., 2018). Previous studies have also highlighted the importance of vegetation type in shaping microbial communities (Cheng et al., 2020).

The β-diversity PCoA analysis revealed the bacterial community structure in both forest types showed a weak response to nitrogen addition, whereas the fungal community structure exhibited a stronger response, particularly in the *P. yunnanensis* Franch. forest, where the response to nitrogen addition was most pronounced (Figure 4). Generally, fungi demonstrate greater resilience to environmental fluctuations and higher tolerance to adverse conditions compared to bacteria (Jia et al., 2020; Liu et al., 2021). Soil fungi are less sensitive to changes in pH, and their diversity typically shows minimal response to nitrogen addition (Wang X. et al., 2023). However, the findings of this study contradict hypothesis (2), as the fungal community structure responded more significantly to nitrogen addition than the bacterial community structure. Similar results have been reported in other studies (Wang et al., 2021). This discrepancy may be attributed to differences in physiological structure and cellular characteristics between fungi and bacteria. We observed that nitrogen addition led to an increase in soil organic matter (SOM) content in *P. yunnanensis* Franch. forests. Studies have reported that, compared to soil bacteria, soil fungi are more sensitive to the decomposition of soil organic matter and the soil carbon cycle. Due to their thicker and interconnected chitinous cell walls, soil fungi are better adapted to a wider range of soil pH variations. This adaptability may result in higher microbial carbon assimilation efficiency under nitrogen addition, allowing soil fungi to utilize soil resources more effectively (Fang et al., 2018; Zhang et al., 2018; Yang et al., 2022). Furthermore, the pronounced wet and dry seasons in the subtropical regions of southern China lead to significant seasonal variations in soil microbial communities and abiotic properties (Zhou et al., 2011), making seasonal fluctuations an important factor that should not be overlooked. Previous studies have confirmed that fungal communities are more sensitive to nitrogen addition and moisture-rich conditions compared to bacterial communities, which is consistent with our findings. This difference may be attributed to significant physiological and cellular structural differences between fungi and bacteria. Fungi, being larger and primarily distributed in large soil pores, are more sensitive to fluctuations in moisture conditions (He et al., 2023). During the wet season, when rainfall and nitrogen addition are abundant, fungal communities are more likely to undergo structural adjustments due to changes in moisture and nutrient availability. Therefore, seasonal variations provide valuable insights into the response mechanisms of microbial communities across different seasons, helping us understand how microbial communities adaptively adjust to enhance their response efficiency to nitrogen inputs under varying environmental conditions. Moreover, increased nitrogen availability may alleviate nitrogen limitation on fungal growth, promoting community changes (Zhao et al., 2020). However, the underlying mechanisms require further investigation in subsequent studies.

### 4.2 Effects of nitrogen addition on the relative abundance and community composition of soil microorganisms

Our results indicated that nitrogen addition did not significantly alter the community composition of soil microorganisms in the *P. yunnanensis* Franch. and *P. armandii* Franch. forests but did significantly influence the relative abundance of specific phyla, contrasting with findings from previous studies. During the wet season, *Acidobacteria* and *Proteobacteria* remained the predominant bacterial phyla in the soil, consistent with our previous findings in the dry season (Hou et al., 2024). Although the bacterial and fungal community compositions in the two forest types were similar, their responses to nitrogen addition were different.

In the *P. yunnanensis* Franch. forest, most bacterial phyla demonstrated a strong buffering capacity to nitrogen addition, showing no significant changes in relative abundance. In contrast, *Basidiomycota* in the fungal community exhibited significant fluctuations, responding notably to nitrogen addition. This response may be linked to changes in litter biomass and substrate input induced by nitrogen addition (Cao et al., 2023). Nitrogen addition significantly increased SOM content in the *P. yunnanensis* Franch. forest. *Basidiomycota*, known for its strong lignin-degrading ability, likely utilized the complex organic compounds in SOM as a nutrient source, promoting its growth and reproduction. Additionally, a significant positive link was found between *Basidiomycota* abundance and soil pH, indicating that higher pH levels may further enhance its relative abundance. The increase in pH likely improved growth conditions for *Basidiomycota*, enabling greater activity in the soil and more efficient decomposition of organic matter.

In the *P. armandii* Franch. forest, *Gemmatimonadetes*, a bacterial phylum unique to the forest, showed a significant response to nitrogen addition. Although research on this group is limited, a significant positive correlation between *Gemmatimonadetes* abundance and pH was observed, with its relative abundance decreasing as pH declined. This trend likely reflects its preference for dry, neutral environments over moist, acidic conditions (DeBruyn et al., 2011). Among the fungal groups, nitrogen addition significantly increased the relative abundance of *Ascomycota* and *Rozellomycota*. *Ascomycota*, a nutrient-rich fungal group, may have benefited from nitrogen addition, which likely altered competitive dynamics among functional groups, favoring nitrogen-tolerant fungi and enhancing their competitive advantage (Morrison et al., 2016). *Rozellomycota*, a recently described fungal phylum, remains poorly understood ecologically, but its unicellular lifestyle and phagocytic nutrient acquisition enable survival in specialized ecological niches (Turner, 2011). Nitrogen addition led to a significant increase in the relative abundance of *Rozellomycota*, which was negatively correlated with pH and positively correlated with NH_4_^+^-N and NO_3_^−^-N. These findings suggest that pH and inorganic nitrogen are critical factors in shaping the growth of *Rozellomycota*, and different fungal groups display distinct preferences for specific soil conditions (Tedersoo et al., 2014). *Rozellomycota* thrives in acidic, nutrient-rich environments.

Microbial co-occurrence networks were constructed using RMT, revealing differences in network properties. In the CK treatment, the bacterial species relationships in the *P. yunnanensis* Franch. forest was less complex than in the *P. armandii* Franch. forest, while fungal species relationships were more complex in the *P. yunnanensis* Franch. forest. However, nitrogen addition increased the number of nodes, edges, and average degree in the bacterial co-occurrence network of the *P. yunnanensis* Franch. forest, reducing the modularity index and increasing network complexity. Nitrogen addition appeared to positively influence the soil bacterial community in the *P. yunnanensis* Franch. forest, possibly due to the increased abundance of key groups (e.g., *Acidobacteria* and *Proteobacteria*), which promote network complexity (Li et al., 2021). The stable ratio of positive correlations suggests that the microbial community in *P. yunnanensis* Franch. forest remains resilient, characterized by cooperative and co-occurring relationships among species. This stability indicates adaptation to specific environmental conditions, with limited sensitivity to external nutrient changes. Network properties in the *P. yunnanensis* Franch. forest peaked at the N20 treatment, suggesting that low nitrogen addition (N10) stimulated bacterial community growth, while higher nitrogen levels (N25) suppressed these positive effects. In contrast, nitrogen addition reduced the complexity of the soil bacterial network in the *P. armandii* Franch. forest, reflecting a negative impact on the bacterial community. This decline may result from the loss of oligotrophic groups (e.g., *Acidobacteria*), which typically obtain nutrients from complex recalcitrant substances (Naylor et al., 2020). Nitrogen input alters the soil carbon-nitrogen balance, stimulates plant growth, increases the supply of fresh carbon sources, and produces unstable, energy-rich carbon substrates (Mason et al., 2023). However, the increase in nutrient availability favors the rapid growth of eutrophic groups, intensifying competition among microorganisms. As eutrophic groups occupy more ecological niches, oligotrophic groups are gradually displaced, leading to their decline and eventual loss (Leff et al., 2015). Nitrogen addition also reduces microbial efficiency in utilizing recalcitrant carbon substrates, further diminishing the abundance of oligotrophic bacteria (Liu et al., 2020). Eutrophic microorganisms, being more competitive under these conditions, promote bacterial independence, weaken inter-microbial interactions, and reduce network connectivity, ultimately decreasing the complexity of the co-occurrence network (Huang et al., 2019). In both forest types, nitrogen addition increased the number of nodes, edges, and the average degree in the soil fungal co-occurrence network, with values peaking at the N10 treatment before declining. This pattern suggests that low nitrogen addition promotes fungal community growth and interaction, whereas high nitrogen addition suppresses these effects, destabilizing the fungal co-occurrence network and reducing its overall stability in both forest types.

### 4.3 Drivers of microbial community response to nitrogen addition

Consistent with hypothesis (3), nitrogen addition affected microbial communities by altering soil properties in both forest types (Shi et al., 2018; Wang et al., 2021). Using the Random Forest model, key factors influencing soil microbial communities were identified as K^+^, pH, NH₄^+^-N, and NO₃^−^-N. Structural equation modeling further revealed that nitrogen addition significantly influenced the α-diversity of bacteria and fungi by modifying soil chemical properties (NH_4_^+^-N, NO_3_^−^-N, K^+^). In the *P. yunnanensis* Franch. forest, NO_3_^−^-N and K^+^ were the main factors regulating soil microbial communities, while NH_4_^+^-N was the key factor in the *P. armandii* Franch. forest. In the *P. yunnanensis* Franch. forest, NO_3_^−^-N negatively affected bacterial community abundance, likely due to its role in stimulating the growth of specific bacterial groups (such as nitrifying and denitrifying bacteria) that utilize nitrate as a substrate. This stimulation intensifies interspecies competition, thereby reducing bacterial community diversity. As previously mentioned, we mentioned that soil pH is influenced by the balance of basic and acidic cations, and structural equation modeling results further exemplify the positive effect of N addition on pH and K^+^. In the *P. yunnanensis* Franch. forest, the increase in pH reduced K^+^ consumption, and nitrogen addition likely enhanced the release of basic cations from litter and roots (Dong et al., 2020). This process improved soil buffering capacity and nutrient availability, creating more favorable conditions for bacterial growth and potentially mitigating competition pressures induced by NO₃^−^-N. Moreover, additional nitrogen input weakened the competition between bacterial and fungal groups. Studies have shown that metabolites produced by the rapid growth and metabolism within different groups can promote energy and material exchange, thus enhancing inter-group interactions (Gralka et al., 2020). Consistently, bacteria were found to positively affect fungi in the *P. yunnanensis* Franch. forest. Whether interactions between microbial communities are cooperative or competitive depends greatly on environmental conditions, such as the rate of resource availability (Ratzke et al., 2020). NH_4_^+^-N had a negative effect on the bacterial community in the *P. armandii* Franch. forest, likely due to its role as a substrate for nitrification. This process enhances soil acidification by lowering pH, which inhibits bacterial growth more than fungal growth (Yang et al., 2024). Additionally, NH_4_^+^-N, preferred by eutrophic groups that utilize reduced nitrogen forms, intensifies interspecies competition (Merrick and Edwards, 1995; Sun et al., 2023). NH_4_^+^-N is a nitrogen source prioritized by many fungi (except for amino acids) (Peng et al., 2022). Fungi can effectively absorb and utilize NH_4_^+^-N, promoting fungal growth and community expansion, subsequently strengthening fungal community dynamics. Notably, the demand for NH_4_^+^-N by bacteria and fungi may exacerbate competition for resources between different communities, with nitrogen inputs reportedly leading to weakened microbial interactions by stimulating the expansion of nitrogen-loving species and competitive exclusion of other species (Zeng et al., 2016).

Differences in the key factors affecting microbial communities in the two stands reflect the species-specific nature of tree species, which not only determine the basic chemical properties of the soil but also, through the regulation of the soil chemical environment, may alter the nutrient sources, metabolic pathways of microorganisms, and their competitive relationships in the community. These findings emphasize that the effects of tree species on soil microbial communities are not limited to changes in physical and chemical environments but, more importantly, how tree species regulate nutrient cycling and microbial competition mechanisms through the inter-root microenvironment, thereby shaping the diversity and function of microbial community structure. This suggests that differences in the response of different tree species to nitrogen deposition may ultimately affect ecosystem function by influencing soil chemistry and microbial interactions.

## 5 Conclusion

Our study demonstrates that species differences drive variations in soil properties between the two forests, but soil microbial communities in both forest types did not exhibit consistent or strong responses to nitrogen addition. Specifically, nitrogen addition did not significantly affect the microbial diversity in the *P. yunnanensis* Franch. and *P. armandii* Franch. forests. However, species differences significantly influenced bacterial diversity. Furthermore, nitrogen addition did not significantly alter the soil microbial community composition in either forest, the fungal community structure in the *P. yunnanensis* Franch. forest underwent significant changes, indicating greater sensitivity of fungal communities to nitrogen deposition compared to bacterial communities. Nitrogen addition increased bacterial network complexity in the *P. yunnanensis* Franch. forest, but reduced network complexity in the *P. armandii* Franch. forest. NO_3_^−^-N and K^+^ played a central role in regulating bacterial and fungal communities in the *P. yunnanensis* Franch. forest, whereas NH_4_^+^-N was the main influencing factor in the *P. armandii* Franch. forest, suggesting that nitrogen addition regulates microbial diversity in both forest soils by modulating nitrogen availability. This study provides a theoretical basis for understanding the response of soil microbes in subtropical coniferous forest ecosystems to nitrogen deposition and highlights the relationship between soil microbial communities and soil properties. Future research should aim to elucidate the mechanisms driving changes in microbial diversity and abundance under nitrogen deposition and explore specific alterations in microbial community structure in response to these environmental shifts.

## Data Availability

The datasets presented in this article are not readily available because they are proprietary and part of ongoing research, and due to confidentiality concerns. Requests to access the datasets should be directed to YS at songyali@swfu.edu.cn.

## References

[ref1] Allison S. D. Hanson C. A. Treseder K. K. (2007). Nitrogen fertilization reduces diversity and alters community structure of active fungi in boreal ecosystems. Soil Biol. Biochem. 39, 1878–1887. doi: 10.1016/j.soilbio.2007.02.001

[ref2] Bao S. D. (2000). Soil and agricultural chemistry analysis. Beijing: China Agricultural Press.

[ref3] Berthrong S. T. Jobbágy E. G. Jackson R. B. (2009). A global meta-analysis of soil exchangeable cations, pH, carbon, and nitrogen with afforestation. Ecol. Appl. 19, 2228–2241. doi: 10.1890/08-1730.1 20014590

[ref4] Cao M. Zheng X. Cui L. Wu F. Gao H. Jiang J. (2023). Soil bacterial communities are more sensitive to short-term nitrogen deposition than fungal communities in subtropical chinese fir forests. Forest Ecol. Manag. 549:121490. doi: 10.1016/j.foreco.2023.121490

[ref5] Chao A. (1984). Non-parametric estimation of the number of classes in a population. Scand. J. Stat. 11, 265–270.

[ref6] Chazdon R. Brancalion P. (2019). Restoring forests as a means to many ends. Science 365, 24–25. doi: 10.1126/science.aax9539, PMID: 31273109

[ref7] Chen S. Chen B. Wang S. Sun L. Shi H. Liu Z.. (2023). Spatiotemporal variations of atmospheric nitrogen deposition in China during 2008–2020. Atmos. Environ. 315:120120. doi: 10.1016/j.atmosenv.2023.120120

[ref8] Chen W. Hou Z. Zhang D. Chen L. Wang K. Song Y. (2024). Increased soil moisture in the wet season alleviates the negative effects of nitrogen deposition on soil microbial communities in subtropical Evergreen broad-leaved Forest. Forests 15:1473. doi: 10.3390/f15081473

[ref9] Chen Y. Zhang Y. Zhang X. Stevens C. Fu S. Feng T.. (2024). Canopy and understory nitrogen additions differently affect soil microbial residual carbon in a temperate forest. Glob. Chang. Biol. 30:e17427. doi: 10.1111/gcb.17427, PMID: 39021313

[ref10] Cheng J. Zhao M. Cong J. Qi Q. Xiao Y. Cong W.. (2020). Soil pH exerts stronger impacts than vegetation type and plant diversity on soil bacterial community composition in subtropical broad-leaved forests. Plant Soil 450, 273–286. doi: 10.1007/s11104-020-04507-2

[ref11] DeBruyn J. M. Nixon L. T. Fawaz M. N. Johnson A. M. Radosevich M. J. A. (2011). Global biogeography and quantitative seasonal dynamics of Gemmatimonadetes in soil. Appl. Environ. Microbiol. 77, 6295–6300. doi: 10.1128/AEM.05005-11, PMID: 21764958 PMC3165389

[ref12] Deng Y. Jiang Y.-H. Yang Y. He Z. Luo F. Zhou J. (2012). Molecular ecological network analyses. BMC Bioinformat. 13, 1–20. doi: 10.1186/1471-2105-13-113, PMID: 22646978 PMC3428680

[ref13] Dong L. Berg B. Sun T. Wang Z. Han X. (2020). Response of fine root decomposition to different forms of N deposition in a temperate grassland. Soil Biol. Biochem. 147:107845. doi: 10.1016/j.soilbio.2020.107845

[ref14] Edgar R. C. (2013). UPARSE: highly accurate OTU sequences from microbial amplicon reads. Nat. Methods 10, 996–998. doi: 10.1038/nmeth.2604, PMID: 23955772

[ref15] Edgar R. C. Haas B. J. Clemente J. C. Quince C. Knight R. (2011). UCHIME improves sensitivity and speed of chimera detection. Bioinformatics 27, 2194–2200. doi: 10.1093/bioinformatics/btr381, PMID: 21700674 PMC3150044

[ref16] Fang Y. Nazaries L. Singh B. K. Singh B. P. (2018). Microbial mechanisms of carbon priming effects revealed during the interaction of crop residue and nutrient inputs in contrasting soils. Glob. Chang. Biol. 24, 2775–2790. doi: 10.1111/gcb.14154, PMID: 29603502

[ref17] Feng K. Peng X. Zhang Z. Gu S. He Q. Shen W.. (2022). iNAP: an integrated network analysis pipeline for microbiome studies. iMeta 1:e13. doi: 10.1002/imt2.13, PMID: 38868563 PMC10989900

[ref18] Finzi A. C. Canham C. D. Van Breemen N. (1998). Canopy tree–soil interactions within temperate forests: species effects on pH and cations. Ecol. Appl. 8, 447–454.

[ref9001] Gardes M. Bruns T. D. (1993). ITS primers with enhanced specificity for basidiomycetes‐application to the identification of mycorrhizae and rusts. Molecular Ecology, 2, 113–118., PMID: 8180733 10.1111/j.1365-294x.1993.tb00005.x

[ref19] Geekiyanage N. (2022). UN decade on ecosystem restoration 2021-2030: are we ready for the grand challenge? Sri Lankan J. Agric. Ecosyst. 4, 1–3. doi: 10.4038/sljae.v4i2.95

[ref20] Gralka M. Szabo R. Stocker R. Cordero O. X. (2020). Trophic interactions and the drivers of microbial community assembly. Curr. Biol. 30, R1176–R1188. doi: 10.1016/j.cub.2020.08.007, PMID: 33022263

[ref21] He D. Guo Z. Shen W. Ren L. Sun D. Yao Q.. (2023). Fungal communities are more sensitive to the simulated environmental changes than bacterial communities in a subtropical forest: the single and interactive effects of nitrogen addition and precipitation seasonality change. Microb. Ecol. 86, 521–535. doi: 10.1007/s00248-022-02092-8, PMID: 35927588

[ref22] Hou Z. Zhang X. Chen W. Liang Z. Wang K. Zhang Y.. (2024). Differential responses of bacterial and fungal community structure in soil to nitrogen deposition in two planted forests in Southwest China in relation to pH. Forests 15:1112. doi: 10.3390/f15071112

[ref23] Hua F. Bruijnzeel L. A. Meli P. Martin P. A. Zhang J. Nakagawa S.. (2022). The ecosystem service and biodiversity contributions and trade-offs of contrasting forest restoration approaches. Science 376, 839–844. doi: 10.1126/science.abl4649 35298279

[ref24] Huang R. McGrath S. P. Hirsch P. R. Clark I. M. Storkey J. Wu L.. (2019). Plant–microbe networks in soil are weakened by century-long use of inorganic fertilizers. Microb. Biotechnol. 12, 1464–1475. doi: 10.1111/1751-7915.13487, PMID: 31536680 PMC6801139

[ref25] Janssens I. A. Luyssaert S. (2009). Nitrogen's carbon bonus. Nat. Geosci. 2, 318–319. doi: 10.1038/ngeo505

[ref26] Jia X. Zhong Y. Liu J. Zhu G. Shangguan Z. Yan W. (2020). Effects of nitrogen enrichment on soil microbial characteristics: from biomass to enzyme activities. Geoderma 366:114256. doi: 10.1016/j.geoderma.2020.114256

[ref27] Kang P. Pan Y. Zhang Y. Gyrat A. Bai H. Yan X. (2024). Soil pathogenic fungal groups and soil nutrient cycling under land use practices in Liupanshan Mountain in China. Land Degrad. Dev. 35, 4781–4794. doi: 10.1002/ldr.5257, PMID: 40007097

[ref28] Kearns P. J. Angell J. H. Howard E. M. Deegan L. A. Stanley R. H. Bowen J. L. (2016). Nutrient enrichment induces dormancy and decreases diversity of active bacteria in salt marsh sediments. Nat. Commun. 7:12881. doi: 10.1038/ncomms12881, PMID: 27666199 PMC5052679

[ref29] Kronzucker H. J. Siddiqi M. Y. Glass A. D. (1997). Conifer root discrimination against soil nitrate and the ecology of forest succession. Nature 385, 59–61. doi: 10.1038/385059a0

[ref30] Leff J. W. Jones S. E. Prober S. M. Barberán A. Borer E. T. Firn J. L.. (2015). Consistent responses of soil microbial communities to elevated nutrient inputs in grasslands across the globe. Proc. Natl. Acad. Sci. 112, 10967–10972. doi: 10.1073/pnas.1508382112, PMID: 26283343 PMC4568213

[ref31] Leng Q. Cui J. Zhou F. Du K. Zhang L. Fu C.. (2018). Wet-only deposition of atmospheric inorganic nitrogen and associated isotopic characteristics in a typical mountain area, southwestern China. Sci. Total Environ. 616-617, 55–63. doi: 10.1016/j.scitotenv.2017.10.240, PMID: 29107779

[ref32] Li B.-B. Roley S. S. Duncan D. S. Guo J. Quensen J. F. Yu H.-Q.. (2021). Long-term excess nitrogen fertilizer increases sensitivity of soil microbial community to seasonal change revealed by ecological network and metagenome analyses. Soil Biol. Biochem. 160:108349. doi: 10.1016/j.soilbio.2021.108349

[ref33] Liu W. Jiang L. Yang S. Wang Z. Tian R. Peng Z.. (2020). Critical transition of soil bacterial diversity and composition triggered by nitrogen enrichment. Ecology 101:e03053. doi: 10.1002/ecy.3053, PMID: 32242918

[ref34] Liu W. Liu L. Yang X. Deng M. Wang Z. Wang P.. (2021). Long-term nitrogen input alters plant and soil bacterial, but not fungal beta diversity in a semiarid grassland. Global Change Biol. 27, 3939–3950. doi: 10.1111/gcb.15681, PMID: 33993594

[ref35] Liu L. Zhang T. Gilliam F. S. Gundersen P. Zhang W. Chen H.. (2013). Interactive effects of nitrogen and phosphorus on soil microbial communities in a tropical forest. PLoS One 8:e61188. doi: 10.1371/journal.pone.0061188, PMID: 23593427 PMC3625167

[ref36] Lladó S. López-Mondéjar R. Baldrian P. (2017). Forest soil bacteria: diversity, involvement in ecosystem processes, and response to global change. Microbiol. Molecular Biol. Rev. 81, 10–1128. doi: 10.1128/MMBR.00063-16 PMC5485800 28404790

[ref37] Logue J. B. Stedmon C. A. Kellerman A. M. Nielsen N. J. Andersson A. F. Laudon H.. (2016). Experimental insights into the importance of aquatic bacterial community composition to the degradation of dissolved organic matter. ISME J. 10, 533–545. doi: 10.1038/ismej.2015.131, PMID: 26296065 PMC4817675

[ref38] Lu X. Mao Q. Gilliam F. S. Luo Y. Mo J. (2014). Nitrogen deposition contributes to soil acidification in tropical ecosystems. Glob. Chang. Biol. 20, 3790–3801. doi: 10.1111/gcb.12665, PMID: 24953639

[ref39] Lu X. Vitousek P. M. Mao Q. Gilliam F. S. Luo Y. Turner B. L.. (2021). Nitrogen deposition accelerates soil carbon sequestration in tropical forests. Proc. Natl. Acad. Sci. 118:e2020790118. doi: 10.1073/pnas.2020790118, PMID: 33846252 PMC8072245

[ref40] Lu Z.-X. Wang P. Ou H.-B. Wei S.-X. Wu L.-C. Jiang Y.. (2022). Effects of different vegetation restoration on soil nutrients, enzyme activities, and microbial communities in degraded karst landscapes in Southwest China. Forest Ecol. Manag. 508:120002. doi: 10.1016/j.foreco.2021.120002

[ref41] Mason A. R. Taylor L. S. DeBruyn J. M. (2023). Microbial ecology of vertebrate decomposition in terrestrial ecosystems. FEMS Microbiol. Ecol. 99:fiad006. doi: 10.1093/femsec/fiad006, PMID: 36631293

[ref42] Merrick M. Edwards R. (1995). Nitrogen control in bacteria. Microbiol. Rev. 59, 604–622. doi: 10.1128/mr.59.4.604-622.1995, PMID: 8531888 PMC239390

[ref43] Morrison E. W. Frey S. D. Sadowsky J. J. van Diepen L. T. Thomas W. K. Pringle A. (2016). Chronic nitrogen additions fundamentally restructure the soil fungal community in a temperate forest. Fungal Ecol. 23, 48–57. doi: 10.1016/j.funeco.2016.05.011

[ref44] Naylor D. Sadler N. Bhattacharjee A. Graham E. B. Anderton C. R. McClure R.. (2020). Soil microbiomes under climate change and implications for carbon cycling. Ann. Rev. Environ. 45, 29–59. doi: 10.1146/annurev-environ-012320-082720

[ref45] Nie Y. Wang M. Zhang W. Ni Z. Hashidoko Y. Shen W. (2018). Ammonium nitrogen content is a dominant predictor of bacterial community composition in an acidic forest soil with exogenous nitrogen enrichment. Sci. Total Environ. 624, 407–415. doi: 10.1016/j.scitotenv.2017.12.142, PMID: 29262382

[ref46] Oksanen J. Blanchet F. G. Kindt R. Legendre P. Minchin P. R. O’hara R.. (2013). Package ‘vegan’. Community ecology package, (Version 2.0) R Foundation for Statistical Computing, Vienna, Austria. Available at: https://cran.r-project.org/web/packages/vegan/vegan.pdf

[ref47] Peng L. Zhang Y. Druzhinina I. S. Kubicek C. P. Wang Y. Zhu Z.. (2022). A facultative ectomycorrhizal association is triggered by organic nitrogen. Curr. Biol. 32, 5235–5249.e7. e5237. doi: 10.1016/j.cub.2022.10.054, PMID: 36402137

[ref48] Quinn Thomas R. Canham C. D. Weathers K. C. Goodale C. L. (2010). Increased tree carbon storage in response to nitrogen deposition in the US. Nat. Geosci. 3, 13–17. doi: 10.1038/ngeo721

[ref49] R ColorBrewer S. Liaw M. A. (2018). Package ‘randomforest’. Berkeley, CA, USA: University of California, Berkeley.

[ref50] Ramirez K. S. Geisen S. Morriën E. Snoek B. L. van der Putten W. H. (2018). Network analyses can advance above-belowground ecology. Trends Plant Sci. 23, 759–768. doi: 10.1016/j.tplants.2018.06.009, PMID: 30072227

[ref51] Ratzke C. Barrere J. Gore J. (2020). Strength of species interactions determines biodiversity and stability in microbial communities. Nat. Ecol. Evolut. 4, 376–383. doi: 10.1038/s41559-020-1099-4, PMID: 32042124

[ref52] Rosenstock N. P. Stendahl J. Van Der Heijden G. Lundin L. McGivney E. Bishop K.. (2019). Base cations in the soil bank: non-exchangeable pools may sustain centuries of net loss to forestry and leaching. Soil 5, 351–366. doi: 10.5194/soil-5-351-2019

[ref53] Shannon C. E. (1948). A mathematical theory of communication. Bell Syst. Tech. J. 27, 379–423. doi: 10.1002/j.1538-7305.1948.tb01338.x

[ref54] Shi L. Zhang H. Liu T. Mao P. Zhang W. Shao Y.. (2018). An increase in precipitation exacerbates negative effects of nitrogen deposition on soil cations and soil microbial communities in a temperate forest. Environ. Pollut. 235, 293–301. doi: 10.1016/j.envpol.2017.12.083 29294455

[ref55] Sokol N. W. Slessarev E. Marschmann G. L. Nicolas A. Blazewicz S. J. Brodie E. L.. (2022). Life and death in the soil microbiome: how ecological processes influence biogeochemistry. Nat. Rev. Microbiol. 20, 415–430. doi: 10.1038/s41579-022-00695-z, PMID: 35228712

[ref56] Song Y. Xing J. Hu C. Song C. Wang Q. Wang S. (2024). Decomposition and carbon and nitrogen releases of twig and leaf litter were inhibited by increased level of nitrogen deposition in a subtropical Evergreen broad-leaved Forest in Southwest China. Forests 15:492. doi: 10.3390/f15030492

[ref57] Sun K. Jiang H.-J. Pan Y.-T. Lu F. Zhu Q. Ma C.-Y.. (2023). Hyphosphere microorganisms facilitate hyphal spreading and root colonization of plant symbiotic fungus in ammonium-enriched soil. ISME J. 17, 1626–1638. doi: 10.1038/s41396-023-01476-z, PMID: 37443341 PMC10504341

[ref58] Svetina D. Rutkowski L. Rutkowski D. (2020). Multiple-group invariance with categorical outcomes using updated guidelines: an illustration using M plus and the lavaan/semtools packages. Struct. Equ. Model. Multidiscip. J. 27, 111–130. doi: 10.1080/10705511.2019.1602776

[ref59] Tedersoo L. Bahram M. Põlme S. Kõljalg U. Yorou N. S. Wijesundera R.. (2014). Global diversity and geography of soil fungi. Science 346:1256688. doi: 10.1126/science.1256688 25430773

[ref60] Terrer C. Vicca S. Stocker B. D. Hungate B. A. Phillips R. P. Reich P. B.. (2018). Ecosystem responses to elevated CO 2 governed by plant–soil interactions and the cost of nitrogen acquisition. New Phytol. 217, 507–522. doi: 10.1111/nph.14872, PMID: 29105765

[ref61] Tian D. Niu S. (2015). A global analysis of soil acidification caused by nitrogen addition. Environ. Res. Lett. 10:024019. doi: 10.1088/1748-9326/10/2/024019

[ref62] Tian Y. Yu M. Xu F. Ouyang S. Xu X. Gao Q.. (2020). Uptake of amino acids and inorganic nitrogen by two dominant temperate grasses. Rhizosphere 14:100199. doi: 10.1016/j.rhisph.2020.100199

[ref63] Turner M. (2011). The evolutionary tree of fungi grows a new branch. Nat. News 11, 2014–1031. doi: 10.1038/news.2011.285, PMID: 39972923

[ref64] Wagg C. Schlaeppi K. Banerjee S. Kuramae E. E. van der Heijden M. G. (2019). Fungal-bacterial diversity and microbiome complexity predict ecosystem functioning. Nat. Commun. 10:4841. doi: 10.1038/s41467-019-12798-y, PMID: 31649246 PMC6813331

[ref65] Wang X. Feng J. Ao G. Qin W. Han M. Shen Y.. (2023). Globally nitrogen addition alters soil microbial community structure, but has minor effects on soil microbial diversity and richness. Soil Biol. Biochem. 179:108982. doi: 10.1016/j.soilbio.2023.108982

[ref66] Wang J. Shi X. Zheng C. Suter H. Huang Z. (2021). Different responses of soil bacterial and fungal communities to nitrogen deposition in a subtropical forest. Sci. Total Environ. 755:142449. doi: 10.1016/j.scitotenv.2020.142449, PMID: 33045514

[ref67] Wang Z. Tao T. Wang H. Chen J. Small G. E. Johnson D.. (2023). Forms of nitrogen inputs regulate the intensity of soil acidification. Glob. Chang. Biol. 29, 4044–4055. doi: 10.1111/gcb.16746, PMID: 37186143

[ref68] White T. J. Bruns T. Lee S. Taylor J. (1990). Amplification and direct sequencing of fungal ribosomal RNA genes for phylogenetics. PCR Protocol 18, 315–322. doi: 10.1016/B978-0-12-372180-8.50042-1, PMID: 40008364

[ref69] Wickham H. (2016). ggplot2: Elegant Graphics for Data Analysis. Springer-Verlag New York, 2016. Available at: https://ggplot2.tidyverse.org

[ref70] Xing J. Hu C. Song C. Wang K. Song Y. (2024). Nitrogen deposition modulates litter decomposition and enhances water retention in subtropical forests. Forests 15:522. doi: 10.3390/f15030522

[ref71] Yang Y. Chen X. Liu L. Li T. Dou Y. Qiao J.. (2022). Nitrogen fertilization weakens the linkage between soil carbon and microbial diversity: a global meta-analysis. Glob. Chang. Biol. 28, 6446–6461. doi: 10.1111/gcb.16361, PMID: 35971768

[ref72] Yang A. Song B. Zhang W. Zhang T. Li X. Wang H.. (2024). Chronic enhanced nitrogen deposition and elevated precipitation jointly benefit soil microbial community in a temperate forest. Soil Biol. Biochem. 193:109397. doi: 10.1016/j.soilbio.2024.109397

[ref73] Yao L. Liu T. Qin J. Jiang H. Yang L. Smith P.. (2024). Carbon sequestration potential of tree planting in China. Nat. Commun. 15:8398. doi: 10.1038/s41467-024-52785-6, PMID: 39333536 PMC11437143

[ref74] Zeng J. Liu X. Song L. Lin X. Zhang H. Shen C.. (2016). Nitrogen fertilization directly affects soil bacterial diversity and indirectly affects bacterial community composition. Soil Biol. Biochem. 92, 41–49. doi: 10.1016/j.soilbio.2015.09.018

[ref75] Zhang T. A. Chen H. Y. Ruan H. (2018). Global negative effects of nitrogen deposition on soil microbes. ISME J. 12, 1817–1825. doi: 10.1038/s41396-018-0096-y, PMID: 29588494 PMC6018792

[ref76] Zhao A. Liu L. Chen B. Fu W. Xie W. Xu T.. (2020). Soil fungal community is more sensitive to nitrogen deposition than increased rainfall in a mixed deciduous forest of China. Soil Ecol. Lett. 2, 20–32. doi: 10.1007/s42832-020-0026-6

[ref77] Zheng M. Zhang T. Luo Y. Liu J. Lu X. Ye Q.. (2022). Temporal patterns of soil carbon emission in tropical forests under long-term nitrogen deposition. Nat. Geosci. 15, 1002–1010. doi: 10.1038/s41561-022-01080-4

[ref78] Zhou G. Wei X. Wu Y. Liu S. Huang Y. Yan J.. (2011). Quantifying the hydrological responses to climate change in an intact forested small watershed in southern China. Glob. Chang. Biol. 17, 3736–3746. doi: 10.1111/j.1365-2486.2011.02499.x

