# Supplementary material for: Differentiated response mechanisms of soil microbial communities to nitrogen deposition driven by tree species variations in subtropical planted forests
Source: Front Microbiol. 2025 Mar 12;16:1534028. doi: 10.3389/fmicb.2025.1534028 (PMC11936929; doi:10.3389/fmicb.2025.1534028)
Supplement: Supplementary file 1 [file Data_Sheet_1.docx]

Supplementary Material

# Supplementary Figures and Tables

## Supplementary Tables

**Table S1**. Response of bacterial and fungal phyla to nitrogen addition in the soil of *Pinus yunnanensis* Franch. forest.

| **Treatments** | **Bacteria** | | | | | | | | | | |  | **Fungi** | | | | | | |
| --- | --- | --- | --- | --- | --- | --- | --- | --- | --- | --- | --- | --- | --- | --- | --- | --- | --- | --- | --- |
|  | **Acidobacteria** | **Proteobacteria** | **Actinobacteria** | **Chloroflexi** | **Verrucomicrobia** | **Planctomycetes** | **Bacteroidetes** | **candidate_division_WPS-2** | **Candidatus_Saccharibacteria** | **unclassified_Bacteria** | **Other** | **Basidiomycota** | **Ascomycota** | **Mortierellomycota** | **Mucoromycota** | **Rozellomycota** | **Chytridiomycota** | **unclassified** | **Other** |
| CK | 33.25(6.23) ^a^ | 30.97(6.16) ^a^ | 5.80(2.10) ^a^ | 7.28(6.18) ^a^ | 3.84(1.26) ^a^ | 4.82(0.48) ^a^ | 2.53(2.68) ^a^ | 1.20(0.33) ^a^ | 0.50(0.44) ^a^ | 8.46(3.19) ^a^ | 1.34(0.64) ^a^ | 39.80(3.27) ^b^ | 30.93(11.47) ^a^ | 22.01(13.49) ^a^ | 4.00(3.14) ^a^ | 0.69(0.19) ^a^ | 1.02(0.89) ^a^ | 1.18(0.77) ^a^ | 0.37(0.29) ^a^ |
| N10 | 34.26(2.44) ^a^ | 31.82(6.84) ^a^ | 6.93(2.00) ^a^ | 4.48(3.93) ^a^ | 4.62(1.45) ^a^ | 3.99(0.96) ^a^ | 1.50(1.34) ^a^ | 1.22(0.13) ^a^ | 0.55(0.51) ^a^ | 8.78(3.04) ^a^ | 1.85(0.84) ^a^ | 45.14(9.17) ^b^ | 27.90(14.91) ^a^ | 23.18(9.84) ^a^ | 1.72(0.70) ^a^ | 0.93(0.65) ^a^ | 0.25(0.29) ^a^ | 0.67(0.18) ^ab^ | 0.21(0.18) ^a^ |
| N20 | 29.80(3.58) ^a^ | 35.70(5.78) ^a^ | 6.63(1.53) ^a^ | 3.42(2.04) ^a^ | 5.30(0.47) ^a^ | 3.94(1.07) ^a^ | 1.33(0.51) ^a^ | 1.28(0.39) ^a^ | 0.61(0.24) ^a^ | 10.50(1.45) ^a^ | 1.40(0.13) ^a^ | 33.79(6.63) ^b^ | 27.78(4.65) ^a^ | 34.25(5.45) ^a^ | 2.12(1.53) ^a^ | 0.86(0.22) ^a^ | 0.39(0.19) ^a^ | 0.31(0.10) ^b^ | 0.49(0.07) ^a^ |
| N25 | 36.92(7.36) ^a^ | 31.00(7.19) ^a^ | 7.42(3.30) ^a^ | 4.86(4.80) ^a^ | 5.01(1.31) ^a^ | 3.23(1.09) ^a^ | 1.02(0.93) ^a^ | 1.05(0.80) ^a^ | 0.42(0.54) ^a^ | 8.01(2.29) ^a^ | 1.06(0.43) ^a^ | 62.96(1.25) ^a^ | 15.33(5.93) ^a^ | 19.10(5.68) ^a^ | 1.30(0.11) ^a^ | 0.32(0.43) ^a^ | 0.17(0.22) ^a^ | 0.66(0.21) ^ab^ | 0.16(0.08) ^a^ |
| N | 0.469 | 0.780 | 0.857 | 0.760 | 0.500 | 0.297 | 0.688 | 0.943 | 0.959 | 0.666 | 0.451 | 0.002** | 0.311 | 0.275 | 0.329 | 0.345 | 0.212 | 0.161 | 0.159 |

a. Data were presented as means (standard errors), and the different letters within columns indicate significant differences among treatments in the same stand (*P* < 0.05).

b. ** indicated *P* < 0.01.

c. Abbreviations: CK, 0 g·N·m^−2^·a^−1^; N10, 10 g·N·m^−2^·a^−1^; N20, 20 g·N·m^−2^·a^−1^; and N25, 25 g·N·m^−2^·a^−1^.

**Table S2.** Correlation of soil bacterial/fungal phylum levels and soil chemical properties in *Pinus* *yunnanensis* Franch. forests.

| **Variable** | **Bacteria** | | | | | | | | | | | | | | | | | | | | | | **Fungi** | | | | | | | | | | | | | | | |
| --- | --- | --- | --- | --- | --- | --- | --- | --- | --- | --- | --- | --- | --- | --- | --- | --- | --- | --- | --- | --- | --- | --- | --- | --- | --- | --- | --- | --- | --- | --- | --- | --- | --- | --- | --- | --- | --- | --- |
|  | **Acidobacteria** | | **Proteobacteria** | | **Actinobacteria** | | **Chloroflexi** | | **Verrucomicrobia** | | **Planctomycetes** | | **Bacteroidetes** | | **candidate_division_WPS-2** | | **Candidatus_Saccharibacteria** | | **unclassified_Bacteria** | | **Other** | | **Basidiomycota** | | **Ascomycota** | | **Mortierellomycota** | | **Mucoromycota** | | **Rozellomycota** | | **Chytridiomycota** | | **unclassified** **_Fungi** | | **Other** | |
|  | ***r*** | ***P*** | ***r*** | ***P*** | ***r*** | ***P*** | ***r*** | ***P*** | ***r*** | ***P*** | ***r*** | ***P*** | ***r*** | ***P*** | ***r*** | ***P*** | ***r*** | ***P*** | ***r*** | ***P*** | ***r*** | ***P*** | ***r*** | ***P*** | ***r*** | ***P*** | ***r*** | ***P*** | ***r*** | ***P*** | ***r*** | ***P*** | ***r*** | ***P*** | ***r*** | ***P*** | ***r*** | ***P*** |
| pH | 0.063 | 0.847 | 0.104 | 0.747 | 0.359 | 0.252 | -0.207 | 0.519 | 0.301 | 0.342 | -0.473 | 0.120 | -0.126 | 0.697 | -0.032 | 0.921 | 0.089 | 0.784 | -0.192 | 0.550 | -0.162 | 0.614 | **0.632** | **0.028** | -0.419 | 0.175 | -0.201 | 0.531 | -0.485 | 0.110 | -0.419 | 0.175 | -0.245 | 0.442 | -0.346 | 0.270 | -0.051 | 0.875 |
| SOM | 0.423 | 0.171 | -0.218 | 0.497 | -0.085 | 0.792 | -0.065 | 0.842 | **0.597** | **0.040** | -0.495 | 0.102 | **-0.578** | **0.049** | -0.390 | 0.210 | -0.365 | 0.244 | 0.281 | 0.377 | -0.354 | 0.258 | 0.471 | 0.122 | -0.514 | 0.087 | 0.114 | 0.723 | -0.426 | 0.168 | -0.224 | 0.483 | **-0.616** | **0.033** | -0.431 | 0.161 | -0.386 | 0.215 |
| NH_4_^+^-N | -0.400 | 0.197 | 0.344 | 0.274 | 0.235 | 0.462 | -0.162 | 0.614 | -0.411 | 0.185 | 0.080 | 0.805 | 0.377 | 0.227 | 0.444 | 0.148 | 0.403 | 0.194 | -0.236 | 0.460 | 0.538 | 0.071 | -0.305 | 0.335 | 0.458 | 0.134 | -0.146 | 0.652 | -0.024 | 0.941 | 0.316 | 0.317 | 0.258 | 0.418 | 0.192 | 0.551 | 0.085 | 0.793 |
| NO_3_^−^-N | -0.300 | 0.344 | 0.447 | 0.145 | 0.543 | 0.068 | **-0.61** | **0.035** | 0.078 | 0.809 | -0.346 | 0.271 | 0.222 | 0.488 | 0.260 | 0.414 | 0.499 | 0.099 | -0.204 | 0.524 | **0.725** | **0.008** | 0.032 | 0.921 | 0.082 | 0.800 | 0.028 | 0.930 | **-0.676** | **0.016** | 0.078 | 0.809 | -0.222 | 0.489 | -0.490 | 0.106 | 0.125 | 0.699 |
| AP | -0.109 | 0.735 | -0.129 | 0.690 | -0.283 | 0.373 | 0.114 | 0.724 | 0.066 | 0.839 | 0.429 | 0.164 | -0.064 | 0.844 | -0.043 | 0.894 | -0.095 | 0.770 | 0.398 | 0.200 | 0.244 | 0.446 | -0.498 | 0.099 | 0.267 | 0.402 | 0.292 | 0.357 | 0.089 | 0.783 | 0.477 | 0.117 | 0.100 | 0.757 | 0.030 | 0.926 | 0.115 | 0.722 |
| K^+^ | -0.068 | 0.834 | 0.136 | 0.673 | 0.054 | 0.867 | -0.068 | 0.834 | 0.282 | 0.374 | -0.316 | 0.317 | -0.211 | 0.511 | -0.025 | 0.938 | -0.023 | 0.944 | 0.115 | 0.721 | -0.434 | 0.158 | 0.117 | 0.718 | -0.232 | 0.468 | 0.156 | 0.629 | -0.111 | 0.730 | -0.274 | 0.389 | -0.158 | 0.624 | -0.291 | 0.360 | 0.177 | 0.583 |
| TN | 0.084 | 0.796 | -0.008 | 0.980 | 0.086 | 0.790 | -0.124 | 0.700 | 0.489 | 0.107 | -0.405 | 0.191 | -0.474 | 0.119 | -0.187 | 0.560 | -0.087 | 0.789 | 0.311 | 0.326 | -0.283 | 0.372 | 0.206 | 0.521 | -0.318 | 0.315 | 0.230 | 0.471 | -0.455 | 0.137 | -0.232 | 0.469 | -0.523 | 0.081 | -0.473 | 0.121 | 0.022 | 0.945 |

Abbreviations: pH, pondus hydrogenii; SOM, soil organic matter; NH_4_^+^-N, Soil ammonium N; NO_3_^−^-N, Soil nitrate N; AP, available phosphorus; K^+^, potassium ion; TN, total nitrogen.

**Table S3.** Response of bacterial and fungal phyla to nitrogen addition in the soil of *Pinus armandii* Franch. forest.

| **Treatments** |  | **Bacteria** | | | | | | | | | | | | | | **Fungi** | | | | | | |
| --- | --- | --- | --- | --- | --- | --- | --- | --- | --- | --- | --- | --- | --- | --- | --- | --- | --- | --- | --- | --- | --- | --- |
|  | **Acidobacteria** | **Proteobacteria** | **Actinobacteria** | **Chloroflexi** | **Verrucomicrobia** | **Planctomycetes** | **Bacteroidetes** | **candidate_division_WPS-2** | **Gemmatimonadetes** | **Candidatus_Saccharibacteria** | **Firmicutes** | **Campilobacterota** | **unclassified_Bacteria** | **Other** | **Basidiomycota** | **Ascomycota** | **Mortierellomycota** | **Mucoromycota** | **Rozellomycota** | **Chytridiomycota** | **unclassified** **_Fungi** | **Other** |
| CK | 24.25(4.06) ^a^ | 33.70(6.26) ^a^ | 8.49(2.66) ^a^ | 2.76(1.47) ^a^ | 3.35(1.09) ^a^ | 3.940(1.28) ^a^ | 1.06(0.63) ^a^ | 0.96(0.15) ^a^ | 1.46(0.25) ^a^ | 0.53(0.45) ^a^ | 0.23(0.12) ^a^ | 0.00(0.00) ^a^ | 18.25(6.08) ^a^ | 1.00(0.29) ^a^ | 59.39(12.49) ^a^ | 20.91(4.83) ^b^ | 16.53(5.70) ^a^ | 1.25(1.42) ^a^ | 0.81(0.42) ^b^ | 0.52(0.26) ^a^ | 0.31(0.17) ^a^ | 0.29(0.16) ^ab^ |
| N10 | 22.65(5.10) ^a^ | 33.52(1.62) ^a^ | 8.03(2.05) ^a^ | 3.24(1.55) ^a^ | 6.40(0.79) ^a^ | 4.02(0.63) ^a^ | 2.03(1.25) ^a^ | 1.84(1.02) ^a^ | 0.94(0.24) ^ab^ | 1.37(1.16) ^a^ | 0.32(0.22) ^a^ | 0.04(0.07) ^a^ | 14.24(2.15) ^a^ | 1.36(0.44) ^a^ | 50.36(12.13) ^a^ | 24.00(9.15) ^b^ | 19.78(12.11) ^a^ | 2.06(2.78) ^a^ | 2.28(0.57) ^b^ | 0.28(0.06) ^a^ | 0.71(0.74) ^a^ | 0.53(0.19) ^a^ |
| N20 | 25.54(9.40) ^a^ | 31.41(4.76) ^a^ | 9.96(4.22) ^a^ | 3.97(0.41) ^a^ | 5.99(2.23) ^a^ | 2.51(0.34) ^a^ | 1.43(1.54) ^a^ | 2.12(1.40) ^a^ | 0.51(0.22) ^b^ | 0.89(0.67) ^a^ | 0.36(0.38) ^a^ | 0.03(0.04) ^a^ | 14.24(2.73) ^a^ | 1.03(0.53) ^a^ | 28.85(5.09) ^b^ | 40.13(4.84) ^a^ | 23.87(8.67) ^a^ | 3.38(4.63) ^a^ | 1.68(0.60) ^b^ | 0.63(0.59) ^a^ | 1.30(1.56) ^a^ | 0.16(0.08) ^b^ |
| N25 | 19.86(10.04) ^a^ | 25.58(10.26) ^a^ | 6.30(0.57) ^a^ | 14.24(18.80) ^a^ | 6.30(4.21) ^a^ | 4.33(1.65) ^a^ | 5.30(8.27) ^a^ | 0.87(0.03) ^a^ | 0.49(0.37) ^b^ | 0.36(0.15) ^a^ | 0.7(0.93) ^a^ | 0.37(0.64) ^a^ | 13.93(3.77) ^a^ | 1.38(0.97) ^a^ | 41.52(4.20) ^ab^ | 23.42(6.09) ^b^ | 28.28(11.73) ^a^ | 1.08(1.52) ^a^ | 4.23(1.35) ^a^ | 0.48(0.20) ^a^ | 0.73(0.57) ^a^ | 0.25(0.20) ^ab^ |
| N | 0.818 | 0.434 | 0.470 | 0.438 | 0.428 | 0.261 | 0.622 | 0.271 | 0.008** | 0.374 | 0.715 | 0.493 | 0.524 | 0.806 | 0.020* | 0.024* | 0.531 | 0.760 | 0.005** | 0.649 | 0.636 | 0.117 |

a. Data were presented as means (standard errors), and the different letters within columns indicate significant differences among treatments in the same stand (*P* < 0.05).

b. * indicated *P* < 0.05, ** indicated *P* < 0.01.

c. Abbreviations: CK, 0 g·N·m^−2^·a^−1^; N10, 10 g·N·m^−2^·a^−1^; N20, 20 g·N·m^−2^·a^−1^; and N25, 25 g·N·m^−2^·a^−1^.

**Table S4.** Correlation of soil bacterial/fungal phylum levels and soil chemical properties in *Pinus armandii* Franch. forests.

| **Variable** | **Bacteria** | | | | | | | | | | | | | | | | | | | | | | | | | | | | **Fungi** | | | | | | | | | | | | | | | |
| --- | --- | --- | --- | --- | --- | --- | --- | --- | --- | --- | --- | --- | --- | --- | --- | --- | --- | --- | --- | --- | --- | --- | --- | --- | --- | --- | --- | --- | --- | --- | --- | --- | --- | --- | --- | --- | --- | --- | --- | --- | --- | --- | --- | --- |
|  | **Acidobacteria** | | **Proteobacteria** | | **Actinobacteria** | | **Chloroflexi** | | **Verrucomicrobia** | | **Planctomycetes** | | **Bacteroidetes** | | **candidate_division_WPS-2** | | **Gemmatimonadetes** | | **Candidatus_Saccharibacteria** | | **Firmicutes** | | **Campilobacterota** | | **unclassified_Bacteria** | | **Other** | | **Basidiomycota** | | **Ascomycota** | | **Mortierellomycota** | | **Mucoromycota** | | **Rozellomycota** | | **Chytridiomycota** | | **unclassified_Fungi** | | **Other** | |
|  | ***r*** | ***r*** | ***r*** | ***P*** | ***r*** | ***P*** | ***r*** | ***P*** | ***r*** | ***P*** | ***r*** | ***P*** | ***r*** | ***P*** | ***r*** | ***P*** | ***r*** | ***P*** | ***r*** | ***P*** | ***r*** | ***P*** | ***r*** | ***P*** | ***r*** | ***P*** | ***r*** | ***P*** | ***r*** | ***P*** | ***r*** | ***P*** | ***r*** | ***P*** | ***r*** | ***P*** | ***r*** | ***P*** | ***r*** | ***P*** | ***r*** | ***P*** | ***r*** | ***P*** |
| pH | 0.075 | 0.817 | 0.302 | 0.340 | 0.090 | 0.780 | -0.238 | 0.457 | -0.534 | 0.074 | 0.108 | 0.738 | -0.197 | 0.540 | -0.238 | 0.457 | **0.836** | **0.001** | -0.053 | 0.870 | -0.225 | 0.482 | -0.224 | 0.485 | 0.420 | 0.174 | -0.131 | 0.685 | **0.691** | **0.013** | -0.402 | 0.195 | -0.462 | 0.131 | -0.112 | 0.729 | **-0.635** | **0.027** | 0.011 | 0.972 | -0.308 | 0.330 | 0.093 | 0.773 |
| SOM | 0.033 | 0.918 | 0.190 | 0.553 | -0.032 | 0.920 | -0.181 | 0.574 | 0.424 | 0.169 | 0.130 | 0.688 | -0.103 | 0.751 | 0.327 | 0.299 | -0.171 | 0.596 | 0.394 | 0.205 | -0.082 | 0.800 | -0.147 | 0.649 | -0.245 | 0.443 | 0.100 | 0.758 | -0.032 | 0.922 | -0.055 | 0.866 | 0.059 | 0.856 | 0.027 | 0.933 | 0.180 | 0.575 | -0.318 | 0.314 | 0.063 | 0.846 | **0.613** | **0.034** |
| NH_4_^+^-N | 0.061 | 0.851 | 0.465 | 0.128 | 0.444 | 0.148 | -0.474 | 0.120 | -0.009 | 0.978 | 0.037 | 0.910 | -0.336 | 0.285 | 0.529 | 0.077 | 0.232 | 0.469 | 0.550 | 0.064 | -0.306 | 0.334 | -0.446 | 0.147 | 0.109 | 0.735 | 0.060 | 0.853 | 0.139 | 0.666 | 0.147 | 0.647 | -0.291 | 0.359 | 0.066 | 0.839 | -0.518 | 0.084 | -0.016 | 0.960 | -0.110 | 0.732 | 0.441 | 0.151 |
| NO_3_^−^-N | -0.120 | 0.711 | -0.406 | 0.190 | -0.184 | 0.568 | 0.358 | 0.254 | 0.345 | 0.272 | 0.056 | 0.863 | 0.261 | 0.413 | -0.106 | 0.742 | **-0.662** | **0.019** | -0.284 | 0.371 | 0.261 | 0.413 | 0.321 | 0.309 | -0.265 | 0.405 | 0.047 | 0.884 | -0.554 | 0.062 | 0.210 | 0.512 | 0.485 | 0.110 | 0.009 | 0.978 | **0.667** | **0.018** | 0.145 | 0.652 | 0.212 | 0.508 | -0.372 | 0.233 |
| AP | -0.147 | 0.649 | -0.328 | 0.298 | -0.084 | 0.796 | 0.303 | 0.338 | 0.307 | 0.332 | 0.125 | 0.699 | 0.218 | 0.495 | 0.008 | 0.980 | **-0.647** | **0.023** | -0.258 | 0.418 | 0.297 | 0.348 | 0.268 | 0.400 | -0.282 | 0.375 | 0.107 | 0.740 | -0.500 | 0.097 | 0.256 | 0.422 | 0.346 | 0.270 | 0.052 | 0.871 | **0.607** | **0.037** | 0.216 | 0.501 | 0.260 | 0.415 | -0.151 | 0.639 |
| K^+^ | -0.003 | 0.994 | 0.217 | 0.498 | -0.032 | 0.922 | -0.184 | 0.566 | 0.245 | 0.443 | 0.095 | 0.769 | -0.074 | 0.819 | 0.282 | 0.375 | 0.071 | 0.827 | 0.485 | 0.110 | -0.106 | 0.742 | -0.140 | 0.664 | -0.164 | 0.610 | 0.158 | 0.624 | 0.153 | 0.635 | -0.121 | 0.708 | -0.129 | 0.690 | 0.076 | 0.813 | 0.007 | 0.983 | -0.392 | 0.207 | 0.023 | 0.944 | **0.638** | **0.026** |
| TN | -0.262 | 0.411 | 0.082 | 0.801 | 0.152 | 0.638 | 0.030 | 0.925 | 0.174 | 0.589 | -0.131 | 0.686 | 0.186 | 0.562 | 0.569 | 0.054 | -0.273 | 0.390 | **0.650** | **0.022** | 0.158 | 0.623 | 0.079 | 0.807 | -0.430 | 0.163 | 0.413 | 0.182 | -0.056 | 0.862 | 0.139 | 0.667 | -0.153 | 0.635 | 0.140 | 0.665 | 0.245 | 0.443 | -0.092 | 0.776 | 0.202 | 0.529 | 0.260 | 0.415 |

Abbreviations: pH, pondus hydrogenii; SOM, soil organic matter; NH_4_^+^-N, Soil ammonium N; NO_3_^−^-N, Soil nitrate N; AP, available phosphorus; K^+^, potassium ion; TN, total nit

## Supplementary Figures


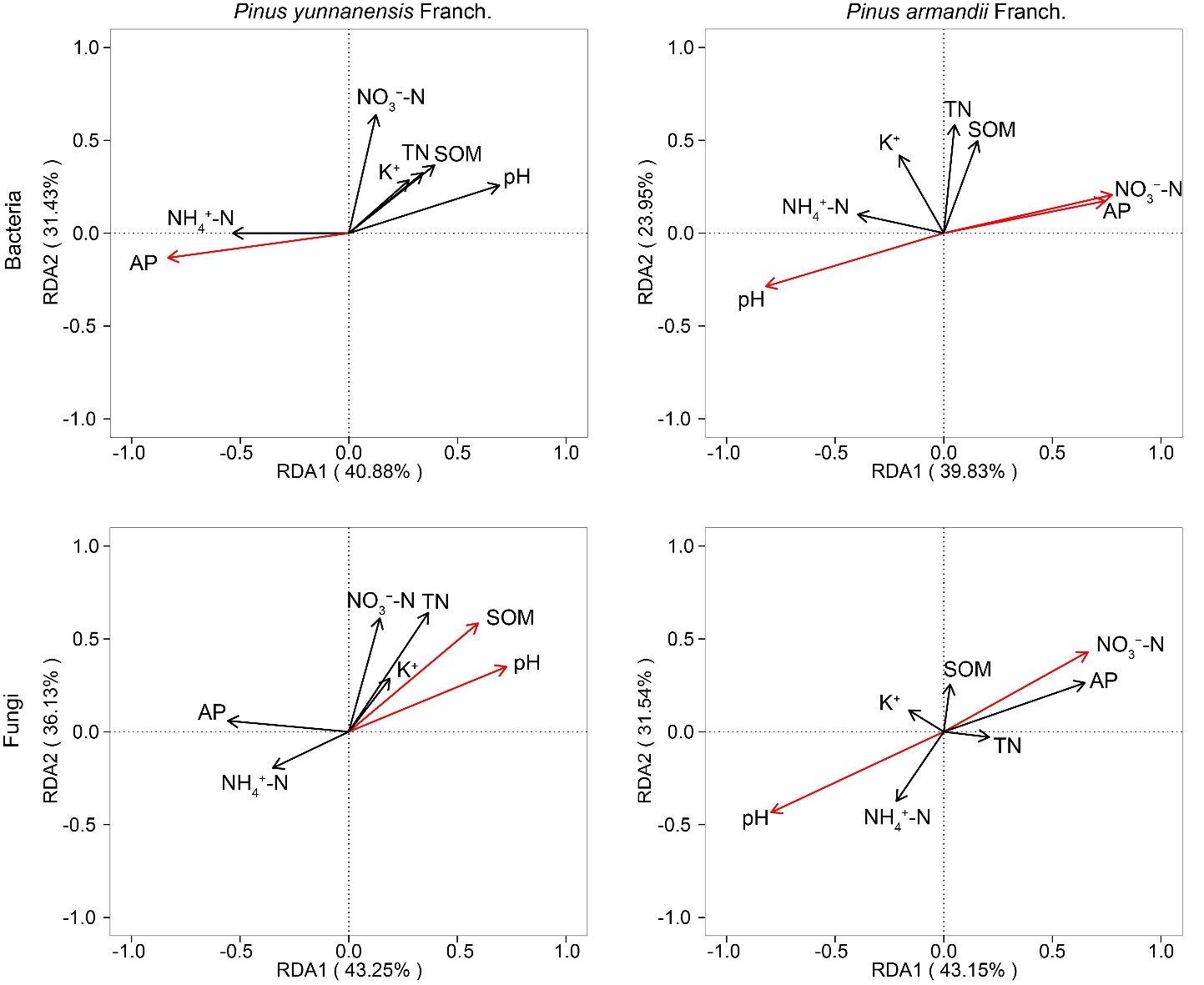


**Figure S1.** RDA analysis of bacterial and fungal community structure in relation to environmental factors. Abbreviations: pH, pondus hydrogenii; SOM, soil organic matter; NH_4_^+^-N, soil ammonium nitrogen; NO_3_^−^-N, soil nitrate nitrogen; TN, total nitrogen; AP, available phosphorus; K^+^, potassium ion. Red arrows indicate significant positive correlations (*P* < 0.05), black arrows indicate non-significant relationships (*P* > 0.05).
